# *Streptococcus dysgalactiae* subsp. *dysgalactiae* isolated from milk of the bovine udder as emerging pathogens: In vitro and in vivo infection of human cells and zebrafish as biological models

**DOI:** 10.1002/mbo3.623

**Published:** 2018-03-25

**Authors:** Cinthia Alves‐Barroco, Catarina Roma‐Rodrigues, Luís R. Raposo, Catarina Brás, Mário Diniz, João Caço, Pedro M. Costa, Ilda Santos‐Sanches, Alexandra R. Fernandes

**Affiliations:** ^1^ Departamento de Ciências da Vida Faculdade de Ciências e Tecnologia UCIBIO Universidade Nova de Lisboa Caparica Portugal; ^2^ Departamento de Química Faculdade de Ciências e Tecnologia UCIBIO Universidade NOVA de Lisboa Caparica Portugal; ^3^ MARE ‐ Marine and Environmental Sciences Centre Departamento de Ciências e Engenharia do Ambiente Faculdade de Ciências e Tecnologia Universidade Nova de Lisboa Caparica Portugal

**Keywords:** bovine, host adhesion/internalization, *Streptococcus dysgalactiae* subsp. *dysgalactiae*, systemic infection, zebrafish

## Abstract

*Streptococcus dysgalactiae* subsp. *dysgalactiae* (SDSD) is a major cause of bovine mastitis and has been regarded as an animal‐restricted pathogen, although rare infections have been described in humans. Previous studies revealed the presence of virulence genes encoded by phages of the human pathogen Group A *Streptococcus pyogenes* (GAS) in SDSD isolated from the milk of bovine udder with mastitis. The isolates SDSD VSD5 and VSD13 could adhere and internalize human primary keratinocyte cells, suggesting a possible human infection potential of bovine isolates. In this work, the in vitro and in vivo potential of SDSD to internalize/adhere human cells of the respiratory track and zebrafish as biological models was evaluated. Our results showed that, in vitro, bovine SDSD strains could interact and internalize human respiratory cell lines and that this internalization was dependent on an active transport mechanism and that, in vivo, SDSD are able to cause invasive infections producing zebrafish morbidity and mortality. The infectious potential of these isolates showed to be isolate‐specific and appeared to be independent of the presence or absence of GAS phage‐encoded virulence genes. Although the infection ability of the bovine SDSD strains was not as strong as the human pathogenic *S*. *pyogenes* in the zebrafish model, results suggested that these SDSD isolates are able to interact with human cells and infect zebrafish, a vertebrate infectious model, emerging as pathogens with zoonotic capability.

## 1 INTRODUCTION

*Streptococcus dysgalactiae* subsp. *dysgalactiae* (SDSD) have been considered exclusively as animal pathogens and are commonly associated with clinical and subclinical bovine mastitis (Abdelsalam, Asheg, & Eissa, 2013; Cervinkova et al., 2013; Rato et al., 2013; Zadoks, Middleton, Mcdougall, Katholm, & Schukken, 2011). In the last years, the association of SDSD with human infections such as upper limb cellulitis in a woman in contact with raw fish (Koh et al., 2009), prosthetic joint infection after knee arthroplasty (Park et al., 2012), and infective endocarditis (Jordal, Glambek, Oppegaard, & Kittang, 2015) has been reported. However, these cases seem to be rare and the role of this subspecies in human pathogenesis remains unclear. It was previously reported the presence of human *S. pyogenes* (GAS) phage virulence genes, namely streptolysin S gene *sagA*, phage‐encoded streptococcal pyrogenic exotoxin genes *speK, speC, speL* and *speM*, phage DNase1 and streptodornase genes *spd1* and *sdn* respectively, located on mobile genetic elements among *S. dysgalactiae* subsp. *dysgalactiae* isolates from bovine mastitis (Rato, Bexiga, Nunes, Vilela, & Santos‐Sanches, 2010; Rato et al., 2011). Generally, genes encoded by mobile genetic elements are preponderant for host invasion and infection (Ribet & Cossart, 2015; Schmidt & Hensel, 2004), suggesting that these GAS genes could play an important role in zoonosis of bovine SDSD.

Recently, we have reported for the first time the ability of milk udder SDSD isolates containing phage encoded GAS genes to adhere and internalize primary human keratinocytes (Roma‐Rodrigues et al., 2016).

As previously reported, zebrafish, *Danio rerio*, show great potential in providing novel information about the pathogenic mechanisms and host responses associated with human streptococcal infections and several zebrafish infection models for the most relevant streptococcal species, the human‐specific *Streptococcus pneumoniae*,* S. pyogenes*, and the zoonotic *Streptococcus iniae* and *Streptococcus agalactiae* have been described (Borst, Patterson, Lanka, Suyemoto, & Maddox, 2013; Miller & Neely, 2005; Novoa & Figueras, 2012; Patterson et al., 2012; Phelps, Runft, & Neely, 2009; Saralahti & Ramet, 2015; Wu, Zhang, Lu, & Lu, 2010).

Adult zebrafish have well‐developed innate and adaptive immune systems resembling the human, since the latter system evolved prior to the evolutionary divergence between fishes and other vertebrates (Novoa & Figueras, 2012; Saralahti & Ramet, 2015). Together, the characteristics of this animal model accentuate the importance in the study of human diseases, thus helping in the study of the zoonotic potential of animal pathogens (Borst et al., 2013; Miller & Neely, 2005; Neely, 2017; Novoa & Figueras, 2012; Patterson et al., 2012; Phelps et al., 2009; Saralahti & Ramet, 2015; Torraca & Mostowy, 2017; Wu et al., 2010; Yoshida, Frickel, & Mostowy, 2017).

The main objective of this work was to further elucidate the zoonotic potential of bovine SDSD isolates containing phage encoded GAS genes collected in two different time periods, 2002–2003 (SDSD VSD5, VSD9, and VSD13) (Rato et al., 2010, 2011, 2013) and 2011–2013 (VSD21, VSD23, and VSD24) to adhere and internalize human respiratory tract cell lines (Detroit 562: pharynx cell carcinoma line, BTEC: primary bronchial/tracheal epithelial cells and A549: lung adenocarcinoma cell line), a preferential niche of colonization/infection of *S*. *pyogenes*, and to analyze the pathogenicity of those isolates using wild‐type zebrafish as the vertebrate animal model.

## 2 MATERIALS AND METHODS

### 2.1 Ethical statement

The study design followed the international (Directive 2010/63/EU of the European parliament, on the protection of animals used for scientific purposes) and national (Decreto‐Lei nº 113/2013) welfare regulations and guidelines (ARRIVE). The animal assays were previously approved by the Portuguese “Direção Geral de Alimentação e Veterinária (DGAV)” (authorization document 0421/000/000/2013). In addition, two authors have a level C FELASA certification (Federation of European Laboratory Animal Science Associations).

### 2.2 *S. dysgalactiae* subsp. *dysgalactiae* collection and identification

SDSD isolates were collected in two different time periods, 2002–2003 (SDSD VSD5, VSD9, and VSD13) (Rato et al., 2010, 2011, 2013) and 2011–2013 (VSD21, VSD23, and VSD24) at the Faculty of Veterinary Medicine, Lisbon, from animals diagnosed with subclinical and clinical mastitis. The identification of the isolates was performed by traditional phenotypic tests based on colony morphology, hemolysis in blood agar plates, Lancefield serologic groups using the SLIDEX Strepto Plus (Biomérieux, France) and absence of catalase activity as previously described (Rato et al., 2010, 2011, 2013). For DNA isolation, all isolates were grown in Todd‐Hewitt broth (Oxoid Limited, Basingstoke, England) supplemented with 0.5% (w/v) of yeast extract (BD, Franklin Lakes) at 37°C until the mid‐exponential phase was reached (OD_600_ of 0.5–0.6). DNA extraction was performed as previously described (Rato et al., 2010, 2011). DNA was quantified in a Nanodrop Spectrophotometer (ThermoFisher Scientific, USA) and integrity was confirmed by gel electrophoresis (1% (w/v) agarose), and images were captured using the Gel Doc XR system and Quantity One 1‐D analysis software (Bio‐Rad, USA). Polymerase Chain Reaction (PCR) amplification of the 16S rRNA gene was performed to reach definitive identification of all the isolates, using primers designed for gram‐positive bacteria (Takahashi et al., 1997), following by automatic Sanger sequencing of the 1.4 kb PCR product (paid service at STAB‐Vida, Lisbon, Portugal). Sequence analysis was performed using BlastN software (http://www.ncbi.nlm.nih.gov/BLAST/) by comparing Sanger sequences of each isolate with the sequences of all the streptococcal species deposited in the GenBank database (see also Figures S1–S3 and Table S1).

### 2.3 Virulence genes screening (PCR) and expression using reverse‐transcriptase PCR (RT‐PCR)

SDSD isolates DNA was extracted as previously described (Rato et al., 2010, 2011). For RNA extraction, SDSD isolates were grown in Todd‐Hewitt broth (Oxoid Limited, Basingstoke, England) supplemented with 0.5% (w/v) of yeast extract (BD, Franklin Lakes) at 37°C until the mid‐exponential phase was reached (OD_600_ of 0.5–0.6). RNA was extracted using NucleoSpin RNAII kit (Macherey‐Nagel, Dueren, Germany) according to the manufacturer's instructions, followed by the addition of 2 U/μl of DNase I (Applied Biosystems/Ambion). RNA was quantified in a Nanodrop Spectrophotometer (ThermoFisher Scientific, USA) and integrity was confirmed by gel electrophoresis (1% (w/v) agarose), and images were captured using the Gel Doc XR system and Quantity One 1‐D analysis software (Bio‐Rad, USA). The cDNA first‐strand was synthesized from 100 ng of total RNA using SuperScript first strand synthesis system (Invitrogen, Germany) according to manufacturer's instructions.

SDSD isolates were screened for the presence and expression of group A *S. pyogenes* (GAS) virulence genes*: sagA* (streptolysin S), *speC*,* speK*,* speL*,* speM* (encoding superantigens), *spd1* (DNase), and *sdn* (streptodornase) by PCR (Rato et al., 2010, 2011). Bovine SDSD VSD13 was used for control purposes. VSD5, VSD9, and VSD13 isolates were previously screened and positive for *sagA* (VSD9), *sdn* and *sagA* (VSD5), and *speC, speK, speL, speM, spd1, sagA,* and *sdn* (VSD13) (Rato et al., 2010, 2011). SDSD isolates were also screened for the presence and expression of genes encoding proteins involved in the adhesion or the internalization process in *Streptococcus dysgalactiae* subspecies *equisimilis* namely *fbpA* (Fibronectin‐binding protein A) and *znuA* (homolog of *adcA S. pyogenes* gene involved in the processes of adhesion and invasion).

The *sagA, speC, speK, speL, speM, spd1, sdn, fbpA,* and *znuA* presence and expression were analyzed using total DNA and cDNA, respectively, as a template. Primer sequences and amplicons expected sizes are listed in Table S2. For each 25 μl PCR reaction mixture, 100 ng of cDNA, 1X reaction buffer for NZYTaq DNA polymerase, 2.5 mmol/L MgCl_2_, 0.4 mmol/L dNTPs NZYMix, 1U NZYTaq DNA polymerase (NZYTech, Lisbon, Portugal) and 1 μmol/L of each primer (ThermoFisher Scientific, Waltham, United States of America) were added. *16S* rRNA gene was used as an endogenous control. The PCR products were analyzed by gel electrophoresis (1% (w/v) agarose). The NZYDNA Ladder VIII (NZYTech, Portugal) was used to estimate DNA fragment size. PCR conditions of *sagA, speC, speK, speL, speM, spd1, sdn* amplification were performed as previously described (Rato et al., 2010, 2011, 2013). PCR conditions of *fbpA* and *znuA* amplification consisted of an initial denaturation cycle (95°C for 5 min) followed by 35 cycles of denaturation (95°C for 30 s), annealing (48°C for *fbpA* or 54°C for *znuA*, for 30 s), and extension (72°C for 45 s). A final extension at 72°C for 7 min was performed. Ultrapure water was used as a negative control in each PCR reaction. *16S* rRNA gene was used as an endogenous control. The PCR products were analyzed by gel electrophoresis (1% (w/v) agarose). The NZYDNA Ladder III (NZYTech, Portugal) was used to estimate DNA fragment size. For *znuA* and *fbpA*, genes, primers were designed using to the available genomic information from *Streptococcus dysgalactiae* subspecies *equisimilis* (SDSE) strains in GenBank and two strains of SDSE COI289 and HSM53 were used as positive controls for PCR.

### 2.4 Antimicrobial resistance patterns

Antimicrobial susceptibility (AMS) tests for SDSD isolated from animals were performed using the disk diffusion technique (Oxoid Ltd, Basingstoke, UK) according to the guidelines from the Clinical and Laboratory Standards Institute (CLSI; http://www.clsi.org/; M31‐A3). The following antimicrobials and doses were selected for testing, based on several criteria: (a) licensing for mastitis treatment in cattle [penicillin (P), pirlimycin (PRL), gentamicin (GEN) and amoxicillin‐clavulanic acid (AMC)]; (b) use in human medicine [erythromycin (ERY), vancomycin (VA), chloramphenicol (CHL), tetracycline (TET)]. For the AMS tests, disks with the following amounts of antibiotics were used: P 10 units (Ref. CT0043B), PRL 2 μg (Ref. CT1668B), GEN 10 μg (Ref. CT0024B), AMC 30 μg (Ref. CT0223), ERY 15 μg (Ref. CT0019B), VA 30 μg (Ref. CT0058B), CHL 30 μg (Ref. CT0013B), and TET 30 μg (Ref. CT0054B) (Oxoid, Basingstoke, United Kingdom). Results were interpreted according to CLSI (M31‐A3).

### 2.5 Determination of minimal inhibitory concentration

Mueller‐Hinton agar (MHA) with 5% sheep blood Broth was supplemented with different concentrations of Gentamicin and Tetracycline (0.5 μg/ml; 1 μg/ml; 2 μg/ml; 4 μg/ml; 8 μg/ml and 16 μg/ml, 64 μg/ml; 125 μg/ml and 250 μg/ml). Higher concentrations of Erythromycin antibiotic were used (8 μg/ml;16 μg/ml; 32 μg/ml; 63 μg/ml; 125 μg/ml; 250 μg/ml; 500 μg/ml; 1000 μg/ml). Direct colony suspension, equivalent to a 0.5 McFarland standard, using colonies from an overnight (18‐ to 20‐hour) sheep blood agar plate and incubated at 37°C, 5% CO_2_, 20–24 hours. *Staphylococcus aureus* ATCC 29213 was used for quality control of determination of minimal inhibitory concentration methods. Results were interpreted according to CLSI (M31‐A3).

### 2.6 Macrolide resistance phenotypes

Resistance only to macrolides (M phenotype) or to macrolides, lincosamides, and streptogramins‐B (MLSB phenotype), either inducible (iMLSB) or constitutive (cMLSB), were evaluated among all streptococcal isolates from this study, by a double‐disk test with erythromycin 15 mg (ERY) and pirlimycin 2 mg (PRL) disks as previously described (Seppala, Nissinen, Yu, & Huovinen, 1993). The discs were placed 15–20 mm apart on Mueller‐Hinton agar, supplemented with 5% (v/v) sheep blood. Inoculation was made with a swab dipped into a bacterial suspension with a turbidity equivalent to a 0–5 McFarland standard. After 24 hours of incubation at 37°C, SDSD bovine showing resistance to erythromycin as well as to clindamycin was defined as having a constitutive type of MLS resistance (cMLS).

### 2.7 Human cell lines and culture conditions

Human cell lines used in the in vitro studies included preferred niches of colonization/infection of *S. pyogenes* from the respiratory track: Detroit 562 (ATCC^®^ CCL138^™^), a cell line derived from the metastatic site of pharynx carcinoma, primary Bronchial/Tracheal Epithelial Cells (BTEC) (ATCC^®^ PCS300010^™^), and A549 (ATCC^®^ CCL185^™^), a cell line derived from a human adenocarcinoma of the alveolar basal epithelial cells. All cell lines were purchased from the American Type Culture Collection (ATCC) (www.atcc.org) and cultured according to the manufacturer′s specifications. Detroit 562 and A549 cultures were maintained in Dulbecco's modified eagle medium (DMEM, ThermoFisher Scientific) supplemented with 10% (v/v) Fetal Bovine serum (ThermoFisher Scientific), and a mixture of 100 U/ml penicillin and 100 μg/ml streptomycin (ThermoFisher Scientific). BTEC were maintained in Airway Epithelial Cell Basal medium (ATCC) supplemented with Bronchial epithelial cell growth kit (ATCC), 33 μmol/l Phenol Red (Sigma) and a mixture of 100 U/ml penicillin and 100 μg/ml streptomycin (ThermoFisher Scientific). For bacterial internalization and adherence assays, human cells were seeded in a 96‐well culture plate (Sigma‐Aldrich Co. LLC, St. Louis, United States of America) at a density of 3 × 10^4^ cells/well and incubated for 24 hours at 37°C, 5% (v/v) CO_2_ and 99% (v/v) relative humidity.

### 2.8 Bacterial internalization and adherence assays

The internalization and adhesion of each streptococcal isolate was performed as previously described (Roma‐Rodrigues et al., 2016) with some modifications. Bacterial isolates were cultivated in 20 ml Todd Hewitt broth supplemented with 0.5% (w/v) yeast extract (THB‐0.5YE) in 100 ml Erlenmeyer flask and grown at 37°C until a standardized optical density at 600 nm (OD_600_) of 0.3–0.4 (5 × 10^7^–1 × 10^8^ cells/ml) was reached. For each isolate, an aliquot of 1 ml of cell suspension was collected and cells were washed three times in fresh THB‐0.5YE and finally resuspended in DMEM. Human cells cultured as described previosuly in a 96‐well cell culture plate were then washed three times with phosphate buffer saline (PBS) and each bacterial suspension added on top of the human cell monolayer (multiplicity of infection of 1:100). Bacterial suspensions were simultaneously serial diluted and plated on Todd‐Hewitt supplemented with 1.5% (w/v) agar (THA) to confirm the initial number of bacteria added to each well (CFU/ml). The 96‐well cell culture plate was then incubated for 2 hours at 37°C, 5% (v/v) CO_2_, and 99% (v/v) relative humidity. A549 cells were also alternatively grown at 4°C for 2 hours. After the incubation period, the supernatant in each well was removed, cells were washed three times with PBS (to remove all extracellular nonadherent bacteria) and used for assessing the number of CFU/ml. Washed cells were then detached and collected from each well through the addition of TrypLE Express Enzyme (ThermoFisher Scientific). After treatment with 0.1% (v/v) Triton X‐100 (Sigma‐Aldrich Co. LLC, St. Louis, United States of America) in PBS to assure lysis of eukaryotic cells, internalized and adherent bacteria were serial diluted and plated on THA to determine CFU/ml [(Adh + int)_value_]. The exact same procedure was performed in human cell free wells in order to obtain procedural negative control values [(Adh + Int)_ctrl_]. The number of total bacteria (final CFU/ml) was estimated using the sum of CFUs counted for the human infected cells (Adh + Int)_value_ and the CFUs of supernatants. In vitro assays were performed using at least three biological replicates and two technical replicates per assay.

Percentage of adherent and internalized Streptococci was calculated with the Equation (1).

(1) $$\begin{array}{cc} \%\;\text{Adhered and Internalized Streptococci}\;= & \frac{(\text{Adh+Int})\text{value}-(\text{Adh+Int})\text{ctrl}}{\Delta f}\\ & \times 100 \end{array}$$

(Adh + Int)value: average CFUs counted for the human infected cells (adhered and internalized); (Adh + Int)ctrl: average CFUs obtained in wells without human cells; ∆*f*: Total CFUs ((Adh+Int)value + average CFUs of supernatants) − number of initial CFUs.

### 2.9 Fluorescence microscopy

For fluorescence microscopy analysis, pelleted bacteria were resuspended in THB‐5YE supplemented with Hoechst 33258 (LifeTechnologies) to a final concentration of 2 μg/ml, incubated at 37°C for 45 min, washed with PBS and resuspended in DMEM. A549 cells grown in 24‐well plate were washed three times with PBS and bacterial cells placed over the human cells. After 2 hour and 4 hour of incubation at 37°C or at 4°C, 5% (v/v) CO_2_, and 99% (v/v) relative humidity, cells were fixed with 2% (w/v) paraformaldehyde for 20 min. at RT, permeabilized with 0.1% (v/v) Triton X‐100 for 5 min and stained with AlexaFluor 488 Phalloidin (LifeTechnologies) according to the manufacturer's instructions. Samples were observed in a Ti‐U Eclipse inverted microscope (Nikon, Tokyo, Japan) with DAPI and FITC fluorescence filter cubes (Nikon) (excitation filter of 340–380 nm and barrier filter at 435–485 nm for DAPI fluorescence filter cube and excitation filter of 465–495 nm and barrier filter at 515–555 nm for FITC fluorescence filter cube). Images were acquired using NIS Elements Basic software (Nikon).

### 2.10 In vivo assays

For the in vivo analysis, SDSD isolates VSD9, VSD13, VSD21, VSD23, and VSD24 were used. *S. pyogenes* GAP58 was used as a positive control. The in vivo animal model used was *Danio rerio* (zebrafish), wild‐type, obtained from national suppliers (Aquaplante, Lisbon, Portugal) and housed at the FCT/UNL fish facilities following the acclimation and experimental conditions described previously (Diniz et al., 2015).

SDSD isolates were grown at 37°C until reach a standardized optical density at 600 nm (OD_600_) of 0.3–0.4 in TSB‐0.5YE. For each isolate, an aliquot of 1 ml of cell suspension was collected and washed three times with fresh TSB‐0.5YE. A 10 μl of the suspension (1 × 10^7^ bacteria (per isolate)) was injected intraperitoneally in a group of 10 wild‐type zebrafish using a Nanofill micro‐syringe (World Precision Instruments, USA). Control groups were injected with 10 μl of TSB‐0.5YE growth medium. Zebrafish groups were then maintained separately in aquaria at a constant temperature of 28°C, using a heated water bath circulator (Haake D1, Haake Messtecknik GmbH Co., Karlsruhe, Germany), without being fed during the 15 days of experiment. Three zebrafish per group were used for histological analysis and were processed while alive (see below). The remaining seven zebrafish per group were used for CFUs assessment after death. To accomplished that, the caudal peduncle (a slice of muscle) and the intraperitoneal region (viscera) were separated, homogenized in PBS (500 μl), and platted (20 μl) in Columbia Blood Agar Base containing 5% sheep blood (Oxoid, Basingstoke, United Kingdom) supplemented with the specific antibiotics. The antibiotics used for supplementation of the medium were selected based on the resistance of the strains in analysis, SDSD VSD13 and VSD21 were grown in medium supplemented with erythromycin (50 μg/ml) (Sigma‐Aldrich Missouri, EUA), SDSD VSD9, VSD21, VSD24 and *S. pyogenes* GAP58 were grown in medium supplemented with tetracycline (10 μg/ml) (Sigma‐Aldrich), SDSD VSD24 was grown in medium supplemented with gentamicin (2 μg/ml) (Sigma‐Aldrich). Fifteen days after injection, the remaining zebrafish were euthanized and processed as described previosuly. The relative CFUs load of each organ was categorized as follows: **0**, no colonies; **1**, 1 to 50 colonies; **2**, 51 to 200 colonies; **3**, 201 to 500 colonies; **4**, 500–700 colonies, **5**, >701 colonies. Four independent experiments were performed for each group of zebrafish (a total of 40 animals per group).

### 2.11 Histological analysis

Whole‐animals were fixed by complete immersion in Davidson's solution (formol—acetic acid—alcohol) for 36 hour (assisted by injection of the fixative intraperitoneally), washed, dehydrated in a graded series of ethanol, infiltrated with xylenes and embedded in paraplast. Sections (5–7 μm thick) were produced longitudinally along the entire body plan to permit a full histopathological screening, with emphasis on the abdominal cavity. Sections were obtained using a Jung RM 2035 microtome (Leica Microsystems). Following deparaffination and rehydration, sections were stained with Hematoxylin (Harris’) and counterstained with Alcoholic Eosin Y (H&E), dehydrated, cleared with xylenes, and mounted with DPX medium. Observations were made with a DMLB model microscope equipped with a DFC 480 digital camera (LeicaMicrosystems) (Costa, 2018).

### 2.12 Identification of the isolates recovered from Zebrafish

The identification of the SDSD and *S. pyogenes* strains was performed by traditional phenotypic tests based on hemolysis in blood agar plates, Lancefield serologic groups using the SLIDEX Strepto Plus (Biomérieux, France) as previously described (Rato et al., 2010, 2011, 2013).

PCR amplification of the *lytR* gene was performed as complementary method for identification of the SDSD isolates. The genome sequence of Lancefield group A *Streptococcus dysgalactiae* subsp. *equisimilis* AC‐2713 (GenBank ID: NC_019042) was used to design primers specific for *lytR* gene (*lytR*‐for 5′ATGAAAATTGGAAAAAAAATA3′ and *lytR*‐rev 5′TTAAGGAAGAGAGGTGGTTGTA3′) (our unpublished results). PCR reactions were performed in a final volume of 25 μl containing: 1 μl of bacterial DNA (0.01 μg), 1X PCR buffer, 2.5 mmol/L of MgCl_2_, 0.4 mmol/L dNTPs, 1U Taq polymerase (NZYTech, Lisbon, Portugal), and 1 μmol/L of each primer (Invitrogen, Auckland, New Zealand). PCR conditions consisted of an initial denaturation cycle (95°C for 5 min) followed by 35 cycles of denaturation (95°C for 30 s), annealing (42°C for 30 s), and extension (72°C for 80 s). A final extension at 72°C for 7 min was also performed. Milli‐Q water was used as a negative control in each PCR reaction. The resulting lytR amplicon, with 1.27 kb, is only amplified in *Streptococcus dysgalactiae*.

The species identification of SDSD and *S. pyogenes* isolates was confirmed by sequencing of 16S rRNA as described previously (Takahashi et al., 1997).

### 2.13 Statistical analysis

GraphPad Prism version 7.0 was used for statistical analysis. Data analyses of in vitro assays were performed using Student's *t* test method. Survival curves of Zebrafish infection assay were analyzed by statistical multiple comparisons performed using Holm‐Sidak method. Statistical significance was considered when *p* < .05.

## 3 RESULTS AND DISCUSSION

### 3.1 Identification and characterization of isolates

SDSD isolates used in this study were collected in two different time periods, 2002–2003 (SDSD VSD5, VSD9, and VSD13) (Rato et al., 2010, 2011, 2013) and 2011–2013 (VSD21, VSD23, and VSD24) at the Faculty of Veterinary Medicine, Lisbon, from animals diagnosed with subclinical and clinical mastitis.

The presumptive identification of isolates recovered between 2011 and 2013 was performed by standard phenotypic tests such as colony morphology, hemolysis in blood agar plates, Lancefield serologic groups and absence of catalase activity. The identification of the bovine mastitis *S. dysgalactiae* subsp. *dysgalactiae* isolates was confirmed using 16S rRNA gene sequencing as described previously (Park et al., 2012). Sequence analysis showed between 99.5% and 99.8% nucleotide identity to *S. dysgalactiae* subsp. *dysgalactiae* strain ATCC 43078 (accession number: NR_115275) deposited in the NCBI database (see Figures S1–S3 and Table S1).

The SDSD strains were tested for the presence of virulence genes by PCR. *S. pyogenes* GAP58, GAP90, and GAP447 isolates and SDSE isolates HSM53 and COI289 were use as controls for the presence of the phage encoded virulence genes and adhesin genes (*fbpA* and *znuA*) respectively (Rato et al., 2011). All strains carry at least one of the virulence genes of *S. pyogenes*. The *sagA* gene was detected in all SDSD isolates (Table 1). VSD9 and VSD23 do not carry any of the other analyzed *S. pyogenes* virulence genes (*speC, speK, speL, speM, spd1* or *sdn*). VSD5 and VSD21 carry *sdn* virulence genes, VSD13 the *speC, speK, speL, speM, and spd1* genes and VSD24 carry the *speC, speK, and spd1* genes (Table 1 and Figure S4). *fbpA* gene was detected in all the SDSD isolates and SDSE control positive strains while the *znuA* gene was observed only in the SDSE strains (Table 1 and Figures S5 and S6). Analysis of the *fbpA* sequences gene revealed 94%–96% homology between the sequences of the SDSD and SDSE strains (Figure S7). A phylogenetic tree of the *fbpA* gene in SDSE and SDSD strains is shown in Figure S8. The transcriptional analysis showed that all expected virulence genes studied were expressed in the respective SDSD isolates carrying them (See Figures S9 and S10).

**Table 1:** Characterization of the bovine SDSD isolates collected and for control SDSE isolates HSME53 and COI289 and control *Streptococcus* *pyogenes* GAP58, GAP90, and GAP447 isolates

Isolate code^a^	Host	Samples Origin	Clinical	Hemolysis	Lancefield	*S*. *pyogenes* virulence genes^b^	Adhesin genes	Resistance phenotype	Reference
VSD5	Bovine	Milk from udder	Subclinical mastitis	α	Group C	*sdn sagA*	*fbpA*	TET	Rato et al., (2010, 2011, 2013)
VSD9	Bovine	Milk from udder	Subclinical mastitis	α	Group C	*sagA*	*fbpA*	TET	
VSD13	Bovine	Milk from udder	Subclinical mastitis	α	Group C	*speC speK speL speM spd1 sagA*	*fbpA*	ERY + TET	
VSD21	Bovine	Milk from udder	Clinical mastitis	α	Group C	*sdn sagA*	*fbpA*	ERY + TET + GEN	This study
VSD23	Bovine	Milk from udder	Clinical mastitis	α	Group C	*sagA*	*fbpA*	TET, GEN	
VSD24	Bovine	Milk from udder	Clinical mastitis	α	Group C	*speC speK spd1 sagA*	*fbpA*	GEN	
HSM53	Human (adult)	Blood	Invasive infection	β	Group G	*—*	*fbpA znuA*	n.d.	Rato et al. (2010, 2011, 2013)
COI289	Human (adult)	Pharyng	Non‐Invasive infection	β	Group G	*—*	*fbpA znuA*	n.d.	
GAP58	Human (adult)	Blood	Invasive infection	β	Group A	*speA speB speF speJ smeZ sagA*	n.d.	TET	Pires et al. (2012)
GAP90	Human (child)	Blood	Invasive infection	β	Group A	*speC speK speL speM spd1, sagA*	n.d.	ERY + TET	
GAP447	Human (adult)	Pharingytis	colonization	β	Group A	*speC speK speL speM spd1, sagA*	n.d.	TET	

a VSD5, VSD9, and VSD13 were collected in 2002–2003 and VSD21, VSD23, VSD24 were collected in 2011–2013.

b *speC, speK, speL, speM—*encode streptococcal pyrogenic exotoxins; *spd1—*DNase; *sdn*—streptodornase (DNase); *fbpA*—Fibronectin‐binding protein A *znuA*, high‐affinity zinc uptake system protein precursor).

Antimicrobial resistance was assessed using standard international methodologies and breakpoints (Clinical and Laboratory Standards Institute, CLSI‐http://clsi.org/) established for beta‐hemolytic streptococci. ERY resistance phenotype was observed only in the VSD21 and VSD13 isolates by disk diffusion method. Accordingly, the analysis of the minimum inhibitory concentrations (MICs) of Erytromicin by serial dilutions revealed that both strains were resistant to high concentrations of erythromycin (Table 2). The VSD21 isolate displayed a MIC between 250 μg/ml and 500 μg/ml and VSD13 growth was not inhibited by any of the erythromycin concentrations tested (MIC > 1000 μg/ml). TET resistance phenotype was observed for VSD5, VSD9, VSD21 VSD23, and GAP58 isolates (resistant to more than 16 μg/ml) (Table 2). GEN resistance phenotype was observed for the three isolates—VSD21, VSD23, and VSD24 by disk diffusion method (diameter of inhibition < 11 mm), however, the minimum inhibitory concentration observed was 4 μg/ml for the VSD24 isolate and 2 μg/ml for the VSD21 and VSD23 isolates (Table 2).

**Table 2:** Resistance profiles and antibiotic minimal inhibitory concentration (MIC) of the SDSD isolates VSD5, VSD9, VSD13, VSD21, VSD23, and VSD24 and for control *S. pyogenes* isolate GAP58

Isolate code^a^	Resistance phenotype	Minimum inhibitory concentration (μg/ml)^b^		
	(by disc difusion)	TET	GEN	ERY
VSD5	TET	64	2	nd
VSD9	TET	16	2	nd
VSD13	TET + cMLS_B_	16	2	>1,000
VSD21	TET+ GEN + cMLS_B_	16	2	250–500
VSD23	TET+ GEN	16	2	nd
VSD24	GEN	4	4	nd
GAP58	TET	16	2	nd

a VSD5, VSD9, and VSD13 were collected in 2002–2003 and VSD21, VSD23, VSD24 were collected in 2011–2013.

b TET, GEN, ERY—Resistant to tetracycline, gentamicin, and erythromycin, respectively. cMLSB only: constitutive MLSB resistance phenotype only; cMLSB + TET: constitutive MLSB resistance phenotype and resistance to tetracycline.

In order to further validate our previous results observed in human keratinocytes (Roma‐Rodrigues et al., 2016), the in vitro interaction/internalization of SDSD isolates in three human cell lines from the respiratory track (a common a niche of colonization/infection of Streptococcus in humans) and the in vivo infection potential in zebrafish of the bovine SDSD isolates was analyzed and correlated with the presence of different virulence factors from human pathogens *S. pyogenes* and SDSE strains in the different SDSD isolates genomes (Table 1) that could be transferred horizontally within *Streptococcus* species (Rato et al., 2010, 2011).

### 3.2 In vitro assays

The interaction of bovine SDSD VSD5, VSD9, VSD13, VSD21, VSD23, and VSD24 was analyzed in human cells derived from a preferred niche of colonization/infection of *S. pyogenes*—the airway system (including pharynx (Detroit 562, pharynx carcinoma), bronchia and trachea (BTEC, primary bronchial/tracheal epithelial cells) and lung (A549, adenocarcinoma human alveolar basal epithelial cells). In the same way invasive human isolates of *S*. *pyogenes* GAP58 and GAP90 and a colonization human isolate of *S*. *pyogenes* GAP447 (Table 1) were also analyzed in these cells.

**Figure 1:**
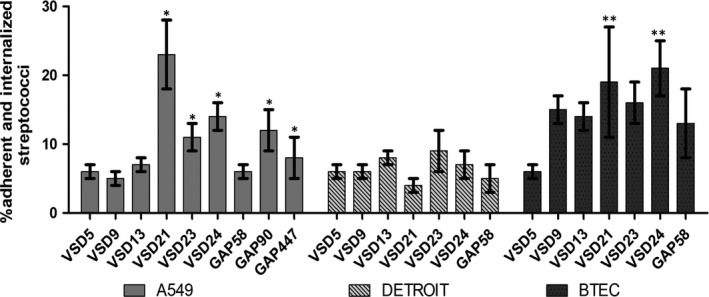
Infection potential of bovine *Streptococcus dysgalactiae* subspecies *dysgalactiae* isolates in human airway stem cell lines. Percentage of adherent and internalized SDSD VSD5, VSD9, VSD13 VSD21, VSD23, VSD24 and *Streptococcus pyogenes* GAP58, GAP90, and GAP447 isolates after the incubation for 2 hours in Detroit 562, A549, and BTEC human cell lines. Represented values are the average value with SEM. *p‐value < .005 for results obtained for A549 cells infection of VSD21, VSD23, VSD24 relative to GAP58. **p‐value < .05 for results obtained for BTEC infection of VSD5 and VSD24 relative to GAP58

Overall, the in vitro results suggested that SDSD strains from bovine mastitis isolated in Portugal from both collections can adhere and internalize Detroit 562, A549, and BTEC human cell lines (Figure 1).

Previously we have reported that compared to SDSD VSD5, SDSD VSD13 had a higher adherence and internalization potential in human primary keratinocytes (Roma‐Rodrigues et al., 2016). This was also observed with airway human cell lines, particularly in primary BTEC (Figure 1). Interestingly, except for SDSD VSD21 in Detroit 562 cell line, SDSD isolates from the 2011–2013 collection (clinical mastitis) have a higher ability to interact with human airway cells compared to the *S*. *pyogenes* invasive isolate GAP58 and SDSD isolates from the 2002–2003 collection (subclinical mastitis) (Figure 1). Comparing the results in *S*. *pyogenes* controls, GAP90 and GAP447 isolates had a significantly higher potential to adhere and colonize A549 cells than *S*. *pyogenes* GAP58 (Figure 1).

Fluorescence microscopy images of A549 cells infected with bovine SDSD VSD9 and SDSD VSD21, as examples, are presented in Figure S11. It is possible to observe bacteria co‐localized with A549 cells. Bacterial cells seem to be surrounded by actin, which suggests that active transport phenomena may be involved. Indeed, incubation for 2 hour of A549 cells with SDSD VSD9 and VSD21 at 4°C showed a marked decrease in the MOI (for VSD9; Figure S12) and in the fluorescence intensity of the observed bacteria in A549 cells (Figure S13) which further supports our hypothesis.

No significant correlation between the number of *S*. *pyogenes* genes and SDSE adhesins present in the genome of the bovine isolates and the capability of the SDSD isolates to interact with human cells was observed (Figure 1 and Table 1). Indeed, SDSD VSD21 harboring only one *S*. *pyogenes* virulence gene (*sagA*), showed a high interaction with A549 lung adenocarcinoma cells and primary BTEC (Figure 1). Moreover, different profiles of interaction with airway human cells were observed between isolates from different collection periods with the same profile of GAS virulent genes (VSD5 and VSD21 with *sagA* and *sdn*, and VSD9 and VSD23 with *sagA*).

Our results indicate that there is no correlation between the expression of the analyzed GAS virulence genes or the *fbpA* and *znuA* adhesin genes in the SDSD isolates and the ability to interact with the airway system human cell lines, suggesting that other mechanisms may be responsible for this ability of SDSD isolates to adhere to and internalize into human cells.

Adherence and invasion of SDSD isolates in epithelial cells has previously documented to occur in mammary cell lines and fish epithelial cell lines cultured in vitro (Calvinho, Almeida, & Oliver, 1998; Calvinho & Oliver, 1998). According to Calvinho and Oliver (1998), the involvement of host kinases, intact microfilaments and de novo eukaryotic protein synthesis are required for the internalization process: a process that appeared to occur by a receptor mediated endocytosis mechanism (Calvinho & Oliver, 1998). Furthermore proteomic studies are ongoing to help to understand the mechanisms involved in interaction between bovine SDSD and human cells (our unpublished results).

### 3.3 Zebrafish infection assays

Since our bovine SDSD isolates (VSD9, VSD13, VSD21, VSD23, and VSD24 (Table 1)) can infect in vitro human cells from the respiratory tract, we questioned if these isolates could infect and trigger an immune system response in other organisms beside the bovine host. For that purpose, the SDSD isolates were inoculated into the well described vertebrate model for the study of infectious diseases, zebrafish (Novoa & Figueras, 2012; Saralahti & Ramet, 2015; Miller & Neely, 2005; Borst et al., 2013; Patterson et al., 2012; Wu et al., 2010; Phelps et al., 2009).

All SDSD isolates were injected intraperitoneally in adult zebrafish. From the total 134 injected zebrafish, 33 zebrafish were injected with sterile TSB, 8 with GAP58 strain, and 93 with the selected SDSD isolates.

**Figure 2:**
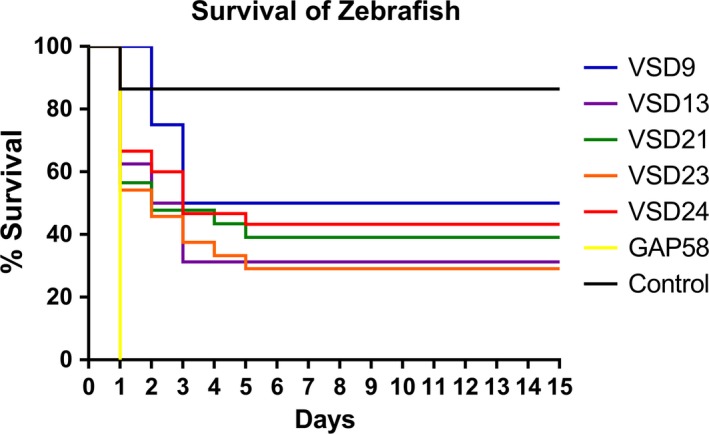
Percentage of survival of zebrafish infected with *S. dysgalactiae* subsp. *dysgalactiae* VSD9 (blue line), VSD13 (purple line), VSD21 (green line), VSD23 (orange line), VSD24 (red line), *S*. *pyogenes* GAP58 (yellow line), and with growth medium TSB‐0.5YE (negative control) (black line). Results are the mean of at least four independent experiments with more than 10 zebrafish in each group and are significantly different with a *p‐value* < .001

**Figure 3:**
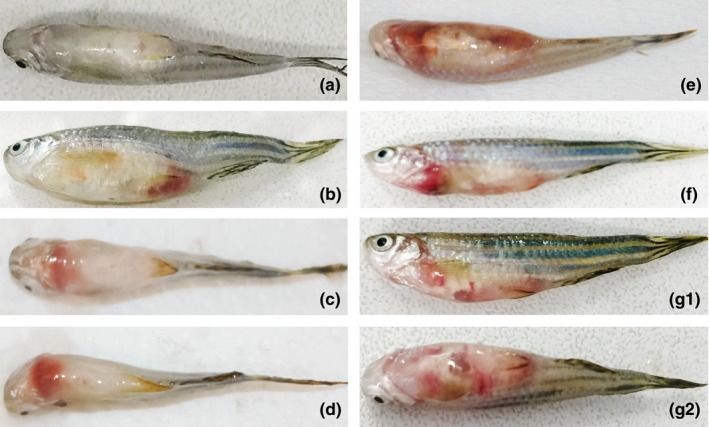
Images of injected zebrafish with (a) no sign of infection/disease (control zebrafish that died 24 hours after inoculation), (b–d) Focal infection (zebrafish injected with SDSD VSD13, VSD24, and VSD21 isolates, respectively), and (e), (f) and (G1; G2) Gross pathology (zebrafish injected intraperitoneally with SDSD VSD21, VSD9, and VSD13, respectively)

The *S*. *pyogenes* GAP58 (Table 1) was used as a positive control since it is an invasive human isolate. Zebrafish injected in the same conditions with TSB medium were used as negative controls. After injection, all isolates (VSD9, VSD13, VSD21, VSD23, VSD24, and GAP58) induced an increased zebrafish mortality compared to the negative control (Figure 2). The human isolate *S*. *pyogenes* GAP58 resulted in an increased mortality compared to SDSD isolates, with 100% mortality after 1 day postinjection (Figure 2). After 5 days of injection with the SDSD isolates, more than 50% of zebrafish had died (Figure 3). Zebrafish injected with *S*. *pyogenes* GAP58 showed the highest mortality, followed by VSD23, VSD13, and VSD21. VSD24 and VSD9 isolates showed the lowest mortality (Figure 2). Interestingly, the results of in vivo assays agree with the in vitro assays with VSD13 (from the first collection) and the SDSD isolates from the second collection with a higher effect (adherence and internalization and mortality) (Figures 1 and 2).

Among the zebrafish that died in the first 5 days after bacterial inoculation, focal infection (Figure 3b–d) and systemic infection (Figure 3e–g) with organ necrosis (data not showed) were observed. For instance, zebrafish infection with VSD13 resulted in both focal and systemic infection (Figure 3b, 3G1, and 3G2, respectively).

The remaining inoculated zebrafish that survived until the end of the experiment (15 days) presented no signs of infection (30% with SDSD VSD13 and VSD23, 40% with SDSD VSD21 and 50% with SDSD VSD24 and VSD9).

The animals that died during the experience or the ones that were euthanized in the end, were recovered, dissected to allow the analysis of the intraperitoneal region (fish viscera) and caudal peduncle (fish muscle) and the number of CFUs assessed. To accomplished this, the homogenates were plated on Columbia Blood Agar Base with 5% Sheep Blood and supplemented with selective antibiotics based on the isolates antibiotic resistance (Table 2; SDSD VSD13 and VSD21 supplemented with erythromycin (50 μg/ml), SDSD VSD9, VSD21, VSD23, and *S*. *pyogenes* GAP58 supplemented with tetracycline (10 μg/ml), and SDSD VSD24 was grown in medium supplemented with gentamicin (2 μg/ml)) to assess the number of CFUs. The confirmation of the presence of SDSD isolates and GAP58 isolate recovered from homogenates of muscles and viscera was performed by phenotypic tests, based on hemolysis in blood agar plates and Lancefield groups. PCR amplification of the *lytR* gene was performed as complementary method for identification of the SDSD isolates. *lytR* gene was detected among isolates recovered from muscles and viscera of the death zebrafish injected with SDSD strains. The species identification of SDSD and *S*. *pyogenes* isolates was confirmed by sequencing of 16S rRNA (Takahashi et al., 1997).

**Figure 4:**
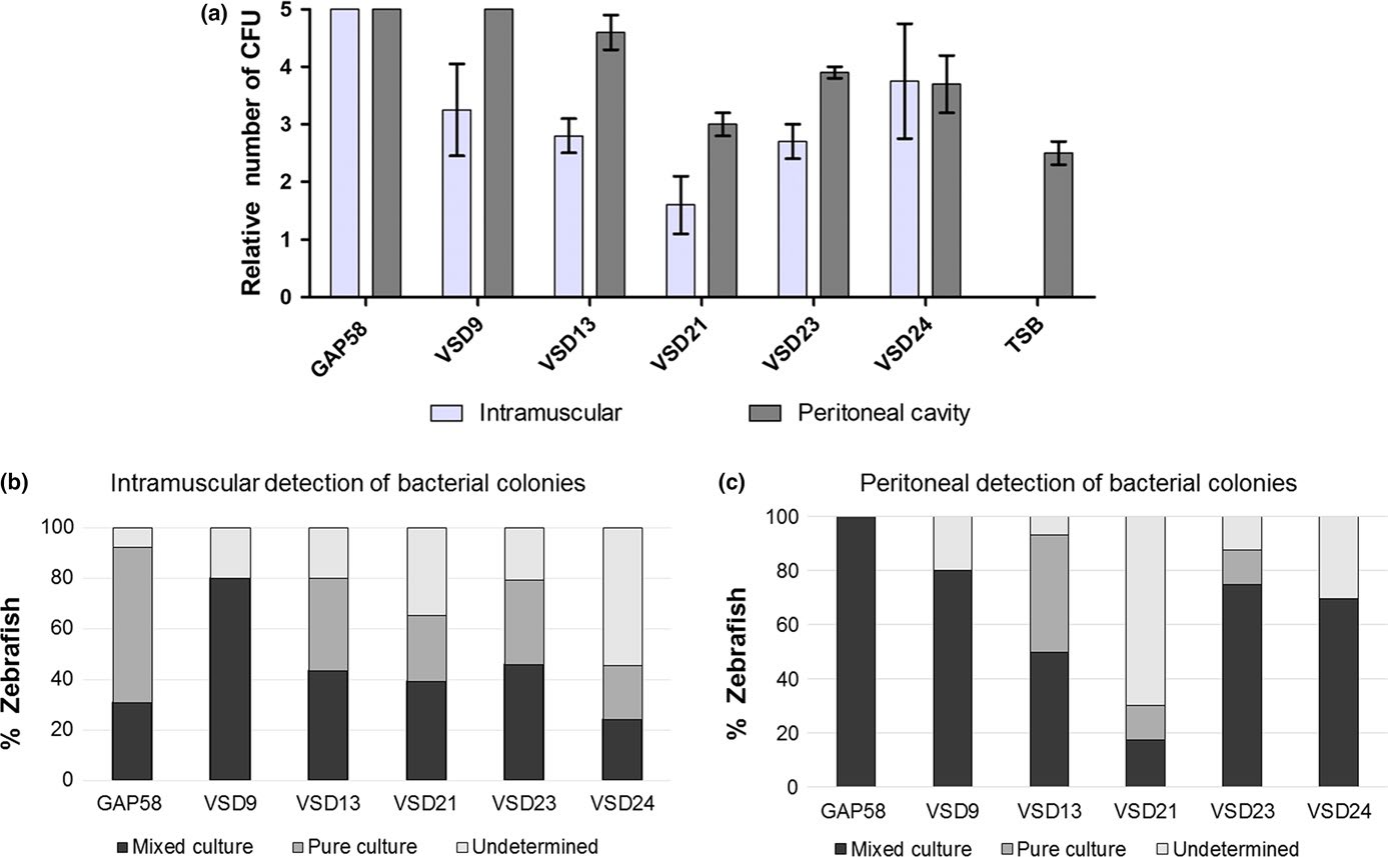
(a) Relative number of CFUs in intramuscular and peritoneal region of zebrafish inoculated with SDSD isolates (VSD13, VSD9, VSD21, VSD23, and VSD24) or *S*. *pyogenes* GAP58. For control purposes, zebrafish were inoculated with 10 μl of TSB‐0.5YE. The relative CFUs in both anatomic regions were scored as follows: 0, no colonies; 1: 1 to 50 colonies; 2: 51 to 200 colonies; 3: 201 to 500 colonies; 4: > 500 colonies. (b) Percentage of zebrafish that died before 15 days of experiment with mixed and pure colonies in the intramuscular region. (c) Percentage of zebrafish that died before 15 days of experiment with mixed and pure colonies in the peritoneal region. Pure culture: containing colonies of the respective SDSD isolate. Mixed culture: contains colonies from more than one type of bacteria

As observed in Figure 4a the bacterial load recovered from the muscles was lower than the bacterial load recovered from the viscera. Interestingly, TET‐resistant, GEN‐resistant and ERY‐resistant mixed cultures (α and β hemolytic) were recovered from both viscera and muscle of zebrafish that died during the experiment (Figure 4b and 4c). The microbiological analysis also allowed to detect the presence of α‐hemolytic SDSD isolates in the viscera (33%) and muscles (5.9%) of the injected zebrafish that were euthanized after 15 days, suggesting a possible colonization ability of the bovine *S. dysgalactiae* subsp. *dysgalactiae* strains in these fish.

The presence of mixed cultures in the muscle suggests that a systemic infection might have occurred, and animal death permitted the spread of other bacteria to this tissue. This can be explained by the presence of α and β‐hemolytic gut bacteria such as *Pseudomonas aeruginosa*, a commensal organism that can cause opportunistic infections in zebrafish (Cantas, Sorby, Alestrom, & Sorum, 2012).

### 3.4 Histological analysis

**Figure 5:**
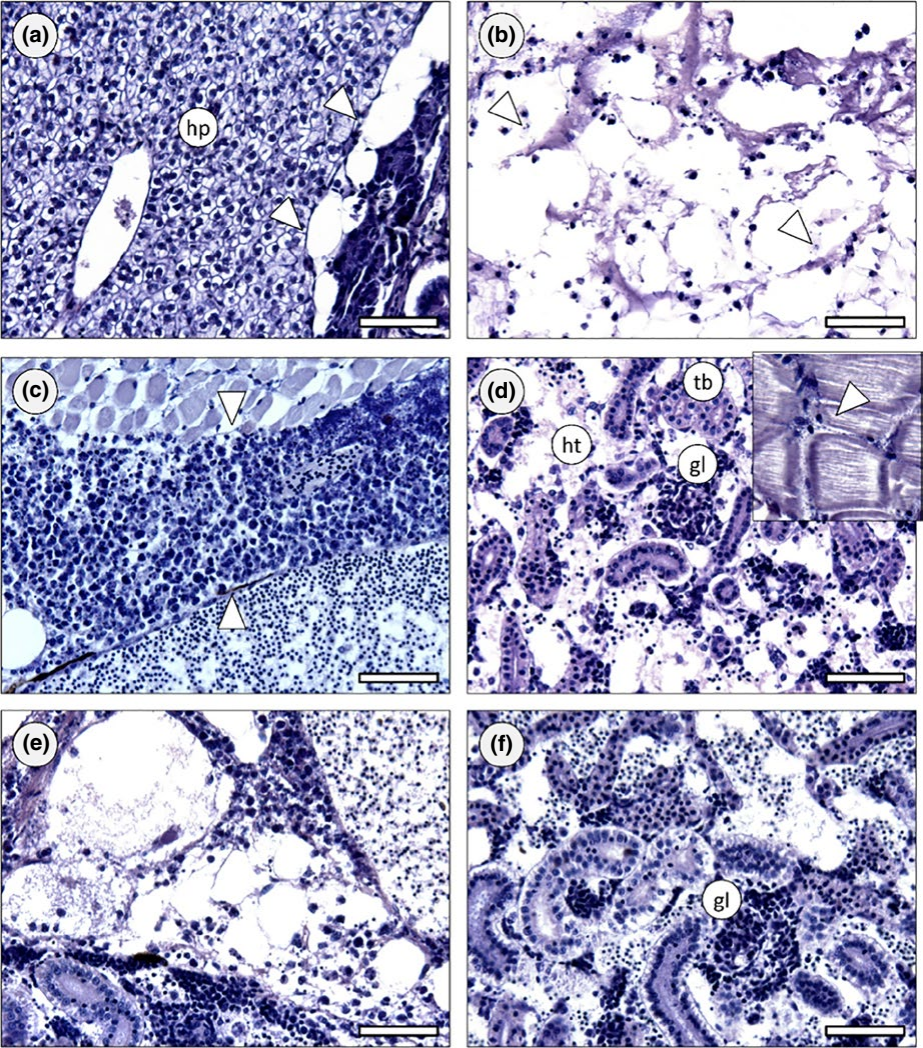
Zebrafish histology (Davidson, H&E). (a) Exemplificative section across the abdominal cavity of a control animal, highlighting hepatic tissue (hp) and interorgan adipose tissue (arrowheads), both devoid of any signs of infection or other pathological features. (b) Section of an animal inoculated with the GAP58 strain, exhibiting severe infection in the interorgan adipose tissue, leading to tissue liquefaction. Cocci can be seen in small clumps of chains, most of which inside the remnants of macrophages (arrowheads). Note that most of the adipose material has been washed‐off during sample processing, leading to potential under evaluation of infection. (c) Massive exudate holding defense cells and bacteria (between arrowheads) in the abdomen of a fish inoculated with the VSD23 strain. The alteration is formed between skeletal muscle and major visceral blood vessel. (d) Severely infected body (trunk) kidney of a zebrafish injected with VSD24 strain. The hematopoietic tissue (ht) shows liquefying necrosis, cocci and macrophage aggregates. Kidney tubules (tb) were obviously less affected but glomeruli (gl) were severely infected as well. *Inset*: Cocci between fascicles of skeletal muscle, with leukocytes infiltrating adjacently. (e) Section across the abdominal cavity of a fish inoculated with the VSD21 strain, showing severe infection in the interorgan adipose tissue and adjacent kidney. (f) Section of a zebrafish treated with the same strain as previous, revealing acute kidney infection affecting haematopoietic tissue and glomeruli, similarly to panel D. Scale bars: 50 μm

All fish inoculated with bacteria showed very significant signs of infection by cocci (Figure 5), especially in the abdominal cavity. These data corroborate the increase in bacterial relative load in the viscera of the fish injected with SDSD and GAP58 when compared to the control (Figure 5a). In most cases, the infection leads to accumulation of exudate, consistent with the distended abdomen observed macroscopically. The exudate contained bacteria, macrophages and monocytes, indicating activation at least of the innate immune system. The infection was particularly noticeable in the adipose tissue around visceral organs, leading to several secondary necrosis of the tissue in most cases (see Figure 5b and compare to control in Figure 5a). Hematopoietic organs were affected in most cases, with emphasis on the kidney (Figure 5d–f). The liver was not significantly affected by infection in any case (even though bacteria could be occasionally found in hepatic blood vessels), as well as the gut. Still, the periphery of the liver exhibit signs of advancing infection. No significant signs of infection were found in other organs or within the circulatory system, even though the animals exhibited signs of diffuse inflammation across the main body plan. Overall, the infection showed signs of spreading from the interorgan space into adjacent organs, which is consistent with the inoculation mode. Bacteria could also be found in skeletal muscle, with variable severity, with a focal pattern but the adverse effects caused by the bacteria in this organ were unclear. The signs of infection were evident and consistent between all treatments, but zebrafish inoculated with the isolate VSD21 revealed the most severe effects, with emphasis on the kidney, adipose tissue and visceral tissue in closer contact with exudate. In this case, the presence of cocci was more evident and widespread throughout the entire abdominal cavity.

The histological evidence suggested that the tested isolates of bacteria can infect wild‐type strains of the zebrafish model and elicit acute and potentially lethal infection, despite the lack of significant evidence for dissemination via blood stream. The effects are consistent between isolates, with the possibility that VSD21 causes the most adverse consequences. The immune system of fish was activated in all cases, with emphasis of massive inflammatory response. Even though the maturation and recruitment of B‐cells in zebrafish remains elusive, the severity of infection in kidney, where B‐cells are mostly produced in this organism (Trede, Langenau, Traver, Look, & Zon, 2004), likely hindered the deployment of adaptive immune response. The dissemination of infection was piecemeal in the abdominal cavity, from the interorgan space to the surface of visceral organs. However, the kidney was the most rapidly and most severely affected organ in all cases. This led to seemingly little compromised hepatic function under the present circumstances of assessment, where other main organs exhibited early failure due to infection, with emphasis on kidney, leading to death. Most of immunity‐related studies with the zebrafish model involving infection has been performed on larval organisms. The current results are accordant with recent studies with the adult model to address *Mycobacterium* inoculation and development of novel vaccines (Oksanen et al., 2013) with respect to efficiency as a model but the present work revealed that *S. dysgalactiae* subsp. *dysgalactiae* infects primarily B‐cell forming tissue, thus hindering adaptive immune responses.

Our results show the ability of bovine SDSD isolates to cause infections in zebrafish. This agrees with previous reports had already shown that SDSD isolates can cause disease in fish (Abdelsalam et al., 2013). These observations may indicate that SDSD strains may have a broader spectrum of hosts than anticipated.

## 4 CONCLUSION

*Streptococcus dysgalactiae* subsp. *dysgalactiae* isolates were found to adhere to and internalize into several human cells and to interact in vivo with the wild‐type, adult, zebrafish model (i.e., with uncompromised immunity), this capability seems to be isolate‐specific and independent of the GAS virulence gene content and the tested adhesins *fbpA* and *znuA*. The findings show that the zebrafish is suitable model to study bacterial pathogens, as it can activate the innate immune system and suffer adverse effects, albeit with differences between strains, which make it an appealing model to study virulence and inflammation. Altogether, the results obtained with the bovine SDSD isolates studied confirm that this subspecies interact with human culture cells in vitro and in vivo can infect the zebrafish model with immune system response. Although the data suggest that these SDSD isolates may have different host preferences, is possible to conclude that SDSD strains are able to infect other hosts than their original one, the cow, and may have a potential zoonotic capability.

## CONFLICT OF INTEREST

None of the authors have any potential conflicts of interest.
