# Supplementary material for: Streptococcus dysgalactiae subsp. dysgalactiae isolated from milk of the bovine udder as emerging pathogens: In vitro and in vivo infection of human cells and zebrafish as biological models
Source: Microbiologyopen. 2018 Mar 25;8(1):e00623. doi: 10.1002/mbo3.623 (PMC6341033; doi:10.1002/mbo3.623)
Supplement: Supplementary file 1 [file MBO3-8-e00623-s001.docx]

# Supporting information

TGCTTGCACCGGTTCAAGGAGTTGCGAACGGGTGAGTAACGCGTAGGTAACCTACCTCATAGCGGGGGATAACTATTGGAAACGATAGCTAATACCGCATGACAATGGAGGACCCATGTCTTTCATTTAAAAGGTGCAACTGCATCACTATGAGATGGACCTGCGTTGTATTAGCTAGTTGGTGAGGTAACGGCTCACCAAGGCGACGATACATAGCCGACCTGAGAGGGTGAACGGCCACACTGGGACTGAGACACGGCCCAGACTCCTACGGGAGGCAGCAGTAGGGAATCTTCGGCAATGGACGGAAGTCTGACCGAGCAACGCCGCGTGAGTGAAGAAGGTTTTCGGATCGTAAAGCTCTGTTGTTAGAGAAGAATGATGGTGGGAGTGGAAAATCCACCATGTGACGGTAACTAACCAGAAAGGGACGGCTAACTACGTGCCAGCAGCCGCGGTAATACGTAGGTCCCGAGCGTTGTCCGGATTTATTGGGCGTAAAGCGAGCGCAGGCGGTTCTTTAAGTCTGAAGTTAAAGGCAGTGGCTCAACCACTGTACGCTTTGGAAACTGGAGAACTTGAGTGCAGAAGGGGAGAGTGGAATTCCATGTGTAGCGGTGAAATGCGTAGATATATGGAGGAACACCGGTGGCGAAAGCGGCTCTCTGGTCTGTAACTGACGCTGAGGCTCGAAAGCGTGGGGAGCAAACAGGATTAGATACCCTGGTAGTCCACGCCGTAAACGATGAGTGCTAGGTGTTAGGCCCTTTCCGGGGCTTAGTGCCGGAGCTAACGCATTAAGCACTCCGCCTGGGGAGTACGACCGCAAGGTTGAAACTCAAAGGAATTGACGGGGGCCCGCACAAGCGGTGGAGCATTGGTTTAATTCGAAGCAACGCGAAGAACCTTACCAGGTCTTGACATCCTCCTGACCGGTCTAGAGATAGACTTTCCCTTCGGGGCAGGAGTGACAGGTGGTGCATGGTTGTCGTCAGCTCGTGTCGTGAGATGTTGGGTTAAGTCCCGCAACGAGCGCAACCCCTATTGTTAGTTGCCATCATTAAGTTGGGCACTCTAACGAGACTGCCGGTAATAAACCGGAGGAAGGTGGGGATGACGTCAAATCATCATGCCCCTTATGACCTGGGCTACACACGTGCTACAATGGTTGGTACAACGAGTCGCAAGCCGGTGACGGCAAGCTAATCTCTTAAAGCCAATCTCAGTTCGGATTGTAGGCTGCAACTCGCCTACATGAAGTCGGAATCGCTAGTAATCGCGGATCAGCACGCCGCGGTGAATACGTTCCCGGGCCTTGTACACACCGCCCGTCACACCACGAGAGTTTGTAACACCCGAAGTCGGTGAGGTAACCTATTAGGAGCCAGCCGCCTAAGGTGGGATAGATGATTGGGGTGAAGTCGTAACAAGG

**Figure S1. *Streptococcus dysgalactiae* subspecies *dysgalactiae* VSDD21 16S ribosomal RNA gene, partial sequence**

TGCTTGCACCGGTTCAAGGAGTTGCGAACGGGTGAGTAACGCGTAGGTAACCTACCTCATAGCGGGGGATAACTATTGGAAACGATAGCTAATACCGCATGACAATGGAGGACCCATGTCTTTCATTTAAAAGGTGCAACTGCATCACTATGAGATGGACCTGCGTTGTATTAGCTAGTTGGTGAGGTAACGGCTCACCAAGGCGACGATACATAGCCGACCTGAGAGGGTGAACGGCCACACTGGGACTGAGACACGGCCCAGACTCCTACGGGAGGCAGCAGTAGGGAATCTTCGGCAATGGACGGAAGTCTGACCGAGCAACGCCGCGTGAGTGAAGAAGGTTTTCGGATCGTAAAGCTCTGTTGTTAGAGAAGAATGATGGTGGGAGTGGAAAATCCACCATGTGACGGTAACTAACCAGAAAGGGACGGCTAACTACGTGCCAGCAGCCGCGGTAATACGTAGGTCCCGAGCGTTGTCCGGATTTATTGGGCGTAAAGCGAGCGCAGGCGGTTCTTTAAGTCTGAAGTTAAAGGCAGTGGCTCAACCACTGTACGCTTTGGAAACTGGAGAACTTGAGTGCAGAAGGGGAGAGTGGAATTCCATGTGTAGCGGTGAAATGCGTAGATATATGGAGGAACACCGGTGGCGAAAGCGGCTCTCTGGTCTGTAACTGACGCTGAGGCTCGAAAGCGTGGGGAGCAAACAGGATTAGATACCCTGGTAGTCCACGCCGTAAACGATGAGTGCTAGGTGTTAGGCCCTTTCCGGGGCTTAGTGCCGGAGCTAACGCATTAAGCACTCCGCCTGGGGAGTACGACCGCAAGGTTGAAACTCAAAGGAATTGACGGGGGCCCGCACAAGCGGTGGAGCATTGGTTTAATTCGAAGCAACGCGAAGAACCTTACCAGGTCTTGACATCCTCCTGACCGGTCTAGAGATAGACTTTCCCTTCGGGGCAGGAGTGACAGGTGGTGCATGGTTGTCGTCAGCTCGTGTCGTGAGATGTTGGGTTAAGTCCCGCAACGAGCGCAACCCCTATTGTTAGTTGCCATCATTAAGTTGGGCACTCTAACGAGACTGCCGGTAATAAACCGGAGGAAGGTGGGGATGACGTCAAATCATCATGCCCCTTATGACCTGGGCTACACACGTGCTACAATGGTTGGTACAACGAGTCGCAAGCCGGTGACGGCAAGCTAATCTCTTAAAGCCAATCTCAGTTCGGATTGTAGGCTGCAACTCGCCTACATGAAGTCGGAATCGCTAGTAATCGCGGATCAGCACGCCGCGGTGAATACGTTCCCGGGCCTTGTACACACCGCCCGTCACACCACGAGAGTTTGTAACACCCGAAGTCGGTGAGGTAACCTATTAGGAGCCAGCCGCCTAAGGTGGGATAGATGATTGGGGTGAAGTCGTAACAAGG

**Figure S2. *Streptococcus dysgalactiae* subspecies *dysgalactiae* VSDD23 16S ribosomal RNA gene, partial sequence**

TGCTTGCACCGGTTCAAGGAGTTGCGAACGGGTGAGTAACGCGTGGGTAACCTACCTCAGATCGGGGGATAACTATTGGAAACGATAGCTAATACCGCATGACAATGGAGGACCCATGTCTTTCATTTAAAAGGTGCTACTGCATCACTATGAGATGGACCTGCGTTGTATTAGCTAGTTGGTGAGGTAACGGCTCACCAAGGCGACGATACATAGCCGACCTGAGAGGGTGAACGGCCACACTGGGACTGAGACACGGCCCAGACTCCTACGGGAGGCAGCAGTAGGGAATCTTCGGCAATGGACGGAAGTCTGACCGAGCAACGCCGCGTGAGTGAAGAAGGTTTTCGGATCGTAAAGCTCTGTTGTTAGAGAAGAATGATGGTGGGAGTGGAAAATCCACCATGTGACGGTAACTAACCAGAAAGGGACGGCTAACTACGTGCCAGCAGCCGCGGTAATACGTAGGTCCCGAGCGTTGTCCGGATTTATTGGGCGTAAAGCGAGCGCAGGCGGTTCTTTAAGTCTGAAGTTAAAGGCAGTGGCTCAACCACTGTACGCTTTGGAAACTGGAGAACTTGAGTGCAGAAGGGGAGAGTGGAATTCCATGTGTAGCGGTGAAATGCGTAGATATATGGAGGAACACCGGTGGCGAAAGCGGCTCTCTGGTCTGTAACTGACGCTGAGGCTCGAAAGCGTGGGGAGCAAACAGGATTAGATACCCTGGTAGTCCACGCCGTAAACGATGAGTGCTAGGTGTTAGGCCCTTTCCGGGGCTTAGTGCCGGAGCTAACGCATTAAGCACTCCGCCTGGGGAGTACGACCGCAAGGTTGAAACTCAAAGGAATTGACGGGGGCCCGCACAAGCGGTGGAGCATTGGTTTAATTCGAAGCAACGCGAAGAACCTTACCAGGTCTTGACATCCTCCTGACCGGTCTAGAGATAGACTTTCCCTTCGGGGCAGGAGTGACAGGTGGTGCATGGTTGTCGTCAGCTCGTGTCGTGAGATGTTGGGTTAAGTCCCGCAACGAGCGCAACCCCTATTGTTAGTTGCCATCATTAAGTTGGGCACTCTAACGAGACTGCCGGTAATAAACCGGAGGAAGGTGGGGATGACGTCAAATCATCATGCCCCTTATGACCTGGGCTACACACGTGCTACAATGGTTGGTACAACGAGTCGCAAGCCGGTGACGGCAAGCTAATCTCTTAAAGCCAATCTCAGTTCGGATTGTAGGCTGCAACTCGCCTACATGAAGTCGGAATCGCTAGTAATCGCGGATCAGCACGCCGCGGTGAATACGTTCCCGGGCCTTGTACACACCGCCCGTCACACCACGAGAGTTTGTAACACCCGAAGTCGGTGAGGTAACCTATTAGGAGCCAGCCGCCTAAGGTGGGATAGATGATTGGGGTGAAGTCGTAACAAGG.

**Figure S3. *Streptococcus dysgalactiae* subspecies *dysgalactiae* VSDD24 16S ribosomal RNA gene, partial sequence**


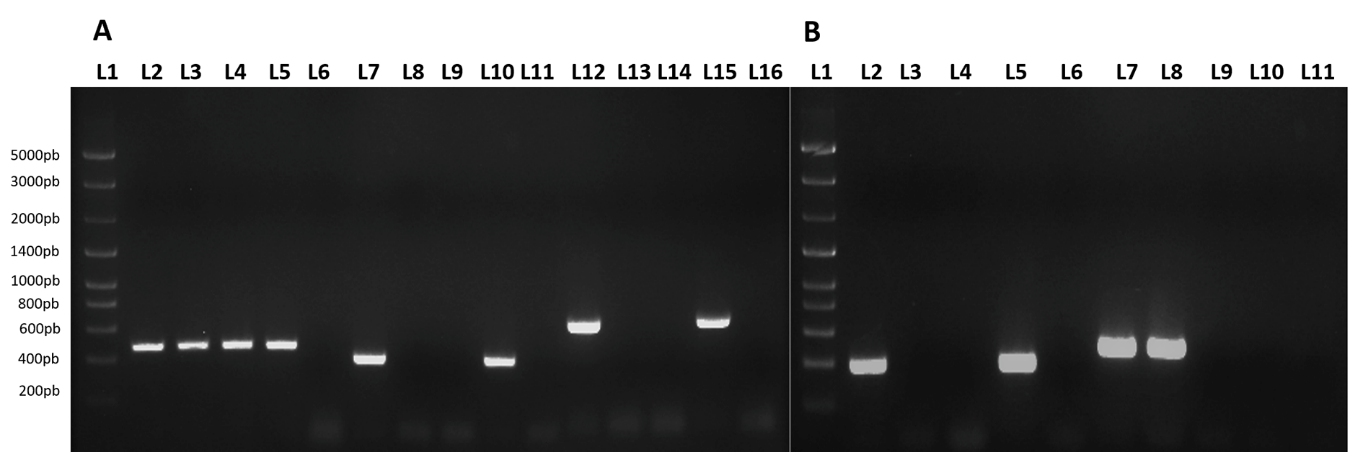


**Figure S4. PCR analysis for the presence of the of *Streptococcus pyogenes* virulence genes** among bovine S. dysgalactiae subsp. *dysgalactiae* (SDSD) isolates. **A) L1**. NZYDNA Ladder VIII (NZYTech, Lisbon, Portugal); **Amplification of sa*gA* gene**: **L2**- VSD13 (positive control); **L3**- VSD21; **L4** - VSD23; **L5** - VSD24; **L6** - Blank (water); **Amplification of *speC* gene**: **L7** - VSD13 (positive control); **L8** - VSD21; **L9** – VSD23; **L10** – VSD24; **L11** - VSDD9 (negative control); **Amplification of *speK* ge**ne: **L12** - VSD13 (positive control); **L13** - VSD21; **L14** – VSD23; **L15** – VSD24; **L16** - VSD9 (negative control); **B) L1**. NZYDNA Ladder VIII; **Amplification of *spd1* gene**: **L2** - VSD13 (positive control); **L3** - VSD21; **L4**- VSD23; **L5** – VSD24; **L6** - VSD9 (negative control); **Amplification of *sdn* gene**: **L7** - VSD5 (positive control); **L8** - VSD21; **L9** – VSD23; **L10** – VSD24 and **L11** - VSD9 (negative control). PCR samples were run on a 1 % (w/v) agarose gel at 80V.





**Figure S5.** **PCR analysis for the presence of the fbpA gene** among bovine S. dysgalactiae subsp. dysgalactiae (SDSD). L1. NZYDNA Ladder III (NZYTech, Lisbon, Portugal); L2-L7: VSD5, VSD9, VSD13, VSD21, VSD23 and VSD24, respectively; L8: COI289 (SDSE); L9: HSM53 (SDSE); L10: blank (water).

**Figure S6.** **Homology analysis of *fbpA* gene sequences** between the sequences of the SDSD and SDSE strains. Homology varied in 94%-96% between sequences.


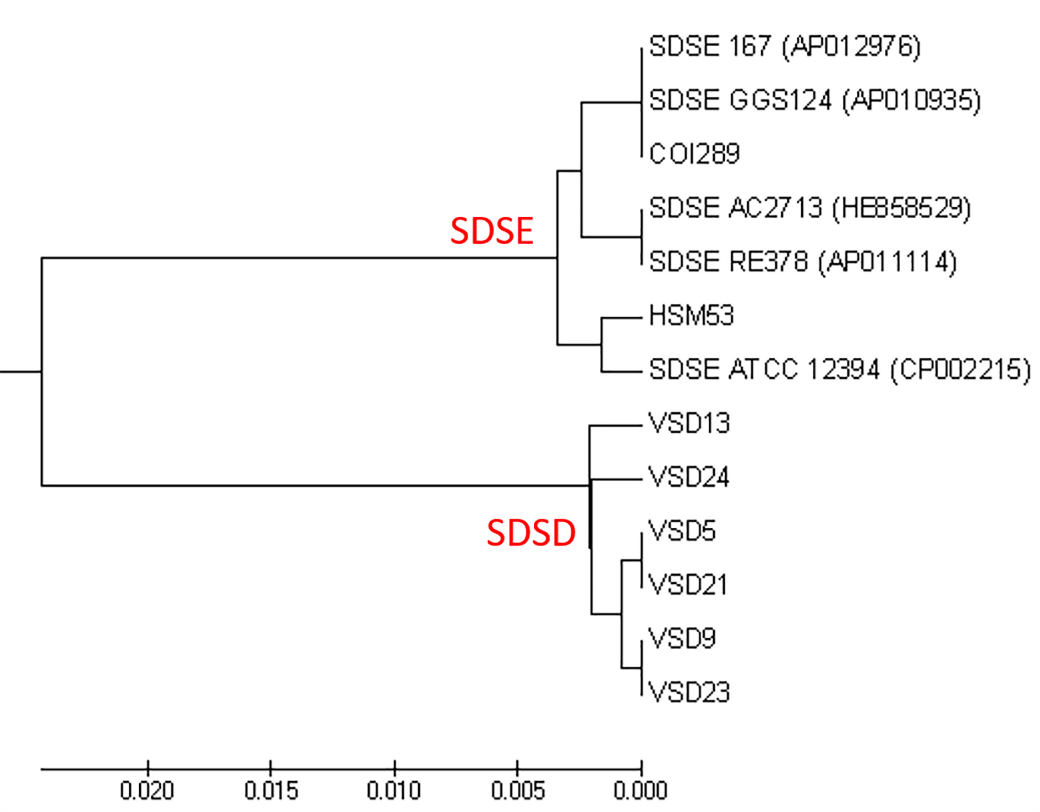


**Figure S7.** **Dendrogram based on UPGMA cluster analysis of *fbpA*-like sequences** of: i) bovine *Streptococcus dysgalactiae* subspecies *dysgalactiae* isolates (SDSD, n= 6); ii) human Streptococcus dysgalactiae subspecies *equisimilis* isolates (SDSE, n= 2); iii) human *Streptococcus dysgalactiae* subspecies *equisimilis* isolates (n= 5) available at the National Centre for Biotechnology Information (NCBI) (Bethesda, MD). GenBank accession numbers for *Streptococcus dysgalactiae* subspecies *equisimilis* isolates are included in parentheses after the gene names. Bootstrap values calculated from 100 replicates. The DNA sequence was analyzed by CLC Genomics Workbench 7.0.4 program.


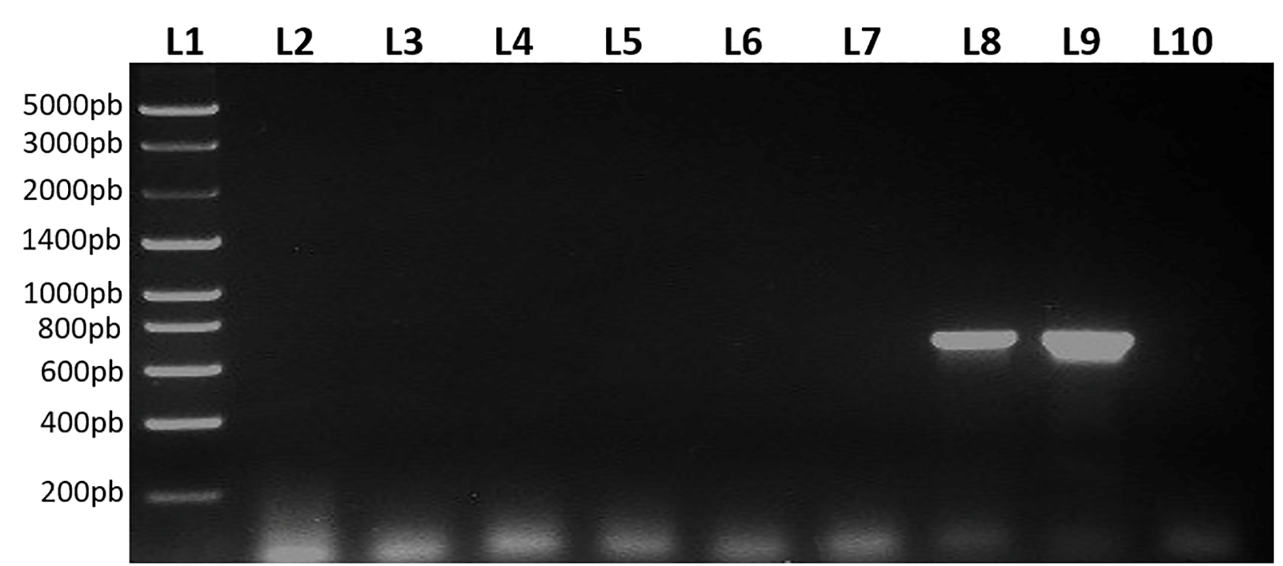


**Figure S8.** **PCR analysis for the presence of the *znuA* gene** among bovine *S. dysgalactiae* subsp. *dysgalactiae* (SDSD). L1. NZYDNA Ladder III (NZYTech, Lisbon, Portugal); L2-L7: VSD5, VSD9, VSD13, VSD21, VSD23 and VSD24, respectively; L8: COI289 (SDSE); L9: HSM53 (SDSE); L10: blank (water).


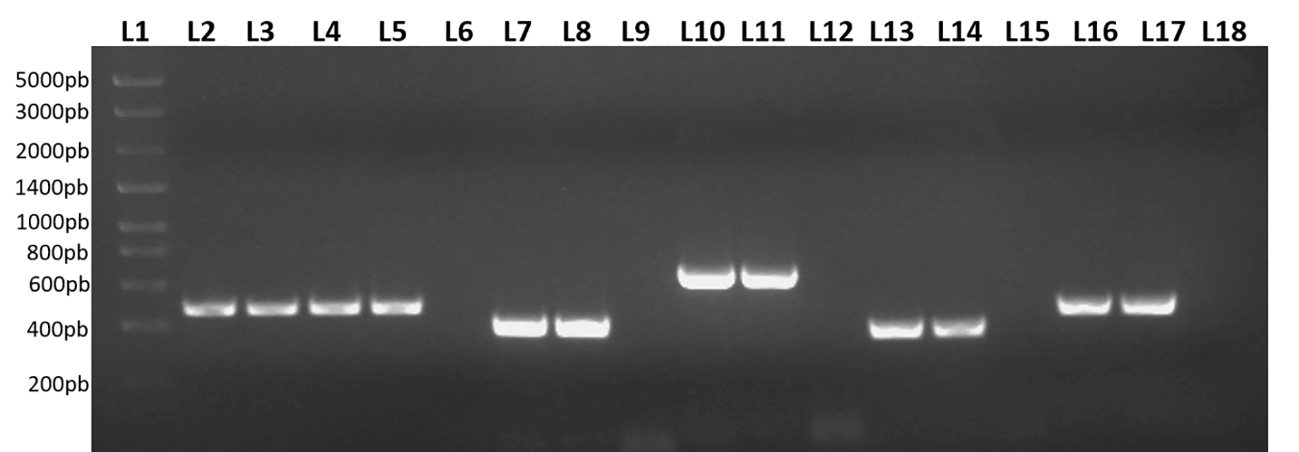


**Figure S9. Gene expression of *Streptococcus pyogenes* virulence genes in *S. dysgalactiae subsp. dysgalactiae* by reverse transcription PCR (RT-PCR).** **L1**. NZYDNA Ladder VIII (NZYTech, Lisbon, Portugal); **Expression of *sagA* gene**: **L2**- VSD13 (positive control); **L3**- VSD21; **L4** - VSD23; **L5** - VSD24 and **L6** - Blank (water); **Expression of *speC* gene**: **L7** - VSD13 (positive control); **L8** - VSD24; **L9** - VSD9 (negative control); **Expression of *speK* ge**ne: **L10** - VSD13 (positive control); **L11** - VSD24; **L12** - VSD9 (negative control); **Expression of *spd1* gene**: **L13** - VSD13 (positive control); **L14** - VSD24; **L15** - VSD9 (negative control); **Expression of *sdn* gene**: **L16** - VSD5 (positive control); **L17** - VSD21 and **L18** - VSD9 (negative control). RT-PCR samples were run on a 1 % (w/v) agarose gel at 80V.





**Fig. S10.** **Gene expression of *fbpA* in *S. dysgalactiae* subsp. *dysgalactiae* by reverse transcription PCR (RT-PCR)**. **L1**. NZYDNA Ladder III (NZYTech, Lisbon, Portugal); **L2:** COI289 (SDSE); **L3:** HSM53 (SDSE); **L4:** blank (water). **L5- L10:** VSD5, VSD9, VSD13, VSD21, VSD23 and VSD24, respectively.


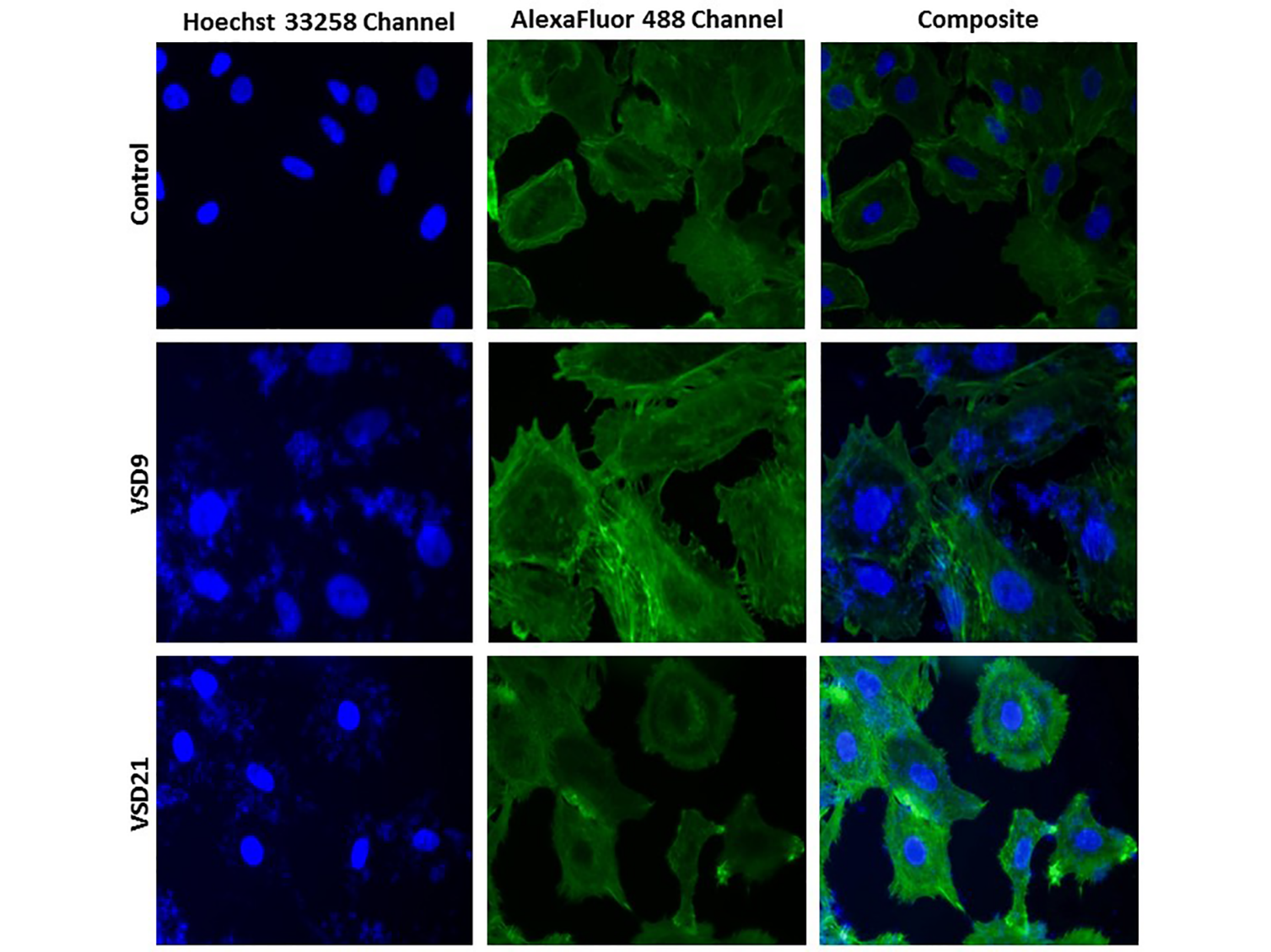


**Figure S11: Representative images of A549 cells incubated for 2 hours at 37ºC with *S*. *dysgalactiae* subspecies *dysgalactiae* isolates VSD9 and VSD21.** Bacterial cells were stained with Hoechst 33258 before infection. A549 cells are stained after infection with AlexaFluor 488 phalloidin. It is possible to observe interaction of bacterial cells with the A459 cells, either in the cell surface or within the eukaryotic cells.





**Figure S12. Percentage of adherent and internalized SDSD VSD9 strain after the incubation for 2 hours at 37ºC and 4ºC in A549 human cell lines.**


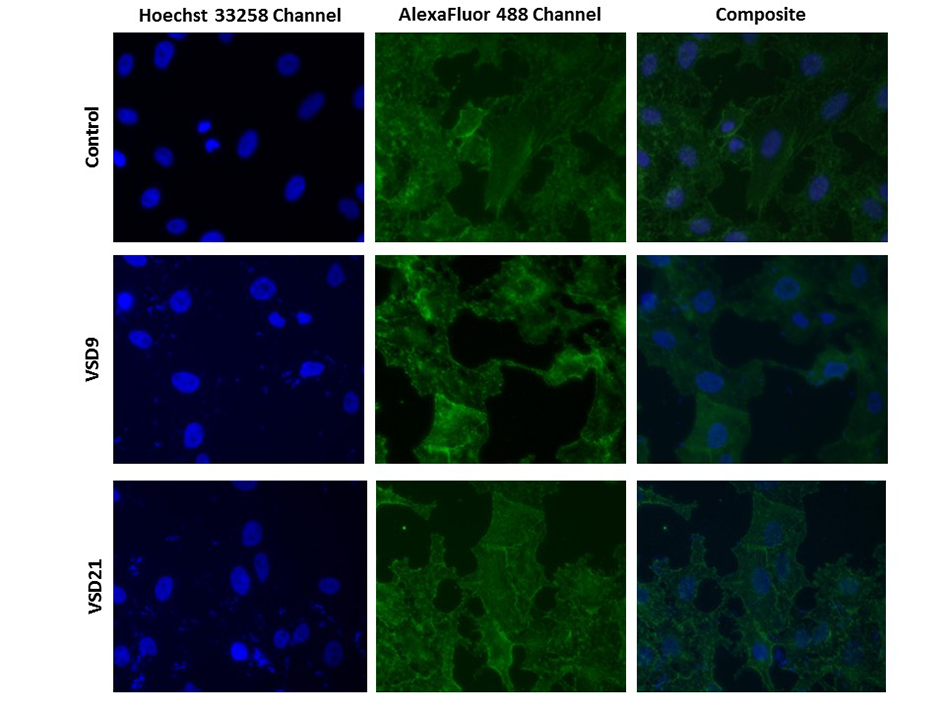


**Figure S13. Representative images of A549 cells incubated for 2 hours at 4°C with *S*. *dysgalactiae* subspecies *dysgalactiae* isolates VSD9 and VSD21. Bacterial cells were stained with Hoechst 33258 before infection. A549 cells are stained after infection with AlexaFluor 488 phalloidin.**

**Table S1. Molecular identification based on homology (>99%) of the 16rRNA of isolates from bovine mastitis isolated in Portugal during 2011-13.**

| **Isolate** | **Hit** | **Description** | **% Identity^a^** | **E-value** | **Score** | **Bit score** | **Gaps** |
| --- | --- | --- | --- | --- | --- | --- | --- |
| **VSD21** | NR_115275 | *Streptococcus dysgalactiae* strain ATCC 43078 16S ribosomal RNA gene, partial sequence | 99.86 | 0 | 2824 | 2547.64 | 1 |
|  | LC269272 | *Streptococcus dysgalactiae* subsp. *dysgalactiae* gene for 16S ribosomal RNA, partial sequence, strain: JCM 5673 | 99.86 | 0 | 2840 | 2562.07 | 1 |
|  | AF015928 | *Streptococcus dysgalactiae* 16S ribosomal RNA gene, partial sequence | 99.86 | 0 | 2826 | 2549.45 | 1 |
|  | AB002484 | *Streptococcus dysgalactiae* DNA for 16S rRNA, strain ATCC 27957 | 99.79 | 0 | 2838 | 2560.27 | 1 |
|  | NR_027517 | *Streptococcus dysgalactiae* strain ATCC 43078 16S ribosomal RNA gene, partial sequence | 99.65 | 0 | 2836 | 2558.46 | 1 |
|  | AB002512 | *Streptococcus dysgalactiae* DNA for 16S rRNA, isolate V26 | 99.51 | 0 | 2826 | 2549.45 | 1 |
| **VSD23** | LC269272 | *Streptococcus dysgalactiae* subsp. *dysgalactiae* gene for 16S ribosomal RNA, partial sequence, strain: JCM 5673 | 99.86 | 0 | 2840 | 2562.07 | 1 |
|  | AF015928 | *Streptococcus dysgalactiae* 16S ribosomal RNA gene, partial sequence | 99.86 | 0 | 2826 | 2549.45 | 1 |
|  | NR_115275 | *Streptococcus dysgalactiae* strain ATCC 43078 16S ribosomal RNA gene, partial sequence | 99.86 | 0 | 2824 | 2547.64 | 1 |
|  | AB002484 | *Streptococcus dysgalactiae* DNA for 16S rRNA, strain ATCC 27957 | 99.79 | 0 | 2838 | 2560.27 | 1 |
|  | NR_027517 | *Streptococcus dysgalactiae* strain ATCC 43078 16S ribosomal RNA gene, partial sequence | 99.65 | 0 | 2836 | 2558.46 | 1 |
|  | AB002512 | *Streptococcus dysgalactiae* DNA for 16S rRNA, isolate V26 | 99.51 | 0 | 2826 | 2549.45 | 1 |
| **VSD24** | NR_115275 | *Streptococcus dysgalactiae* strain ATCC 43078 16S ribosomal RNA gene, partial sequence | 99.58 | 0 | 2804 | 252961 | 1 |
|  | LC269272 | *Streptococcus dysgalactiae* subsp. dysgalactiae gene for 16S ribosomal RNA, partial sequence, strain: JCM 5673 | 99.58 | 0 | 2820 | 2544.04 | 1 |
|  | AF015928 | *Streptococcus dysgalactiae* 16S ribosomal RNA gene, partial sequence | 99.58 | 0 | 2806 | 2531.41 | 1 |
|  | AB002484 | *Streptococcus dysgalactiae* DNA for 16S rRNA, strain ATCC 27957 | 99.51 | 0 | 2818 | 2542.23 | 1 |
|  | NR_027517 | *Streptococcus dysgalactiae* strain ATCC 43078 16S ribosomal RNA gene, partial sequence | 99.37 | 0 | 2816 | 2.540.43 | 1 |
|  | AB002512 | *Streptococcus dysgalactiae* DNA for 16S rRNA, isolate V26 | 99.23 | 0 | 2806 | 2.531.41 | 1 |

^a^ Percentage of homology of the overlapping sequence.

**Table S2. PCR primers used for screening of virulence determinants among group C *Streptococcus dysgalactiae* subsp. *dysgalactiae* and of isolates from bovine mastitis isolated in Portugal during 2011-13.**

| **Primer name** | **Sequence (5’-3’)** | **Product** | **Reference** |
| --- | --- | --- | --- |
| **Pyrogenic exotoxins:** |  |  |  |
| *speC* (for.) | GCAGGGTAAATTTTTCAACGACACACA | 407 | 1 |
| *speC* (rev.) | TGTGCCAATTTCGATTCTGCCGC |  |  |
| *speK* (for.) | TACAAATGATGTTAGAAATCCAAGGAACATATATGCT | 656 | 1 |
| *speK* (rev.) | CAAAGTGACTTACTTTACTCATATCAATCGTTTC |  |  |
| *speL* (for.) | CTGTTAGGATGGTTTCTGCGGAAGAG | 605 | 1 |
| *speL* (rev.) | AGCACCTTCCTCTTTCTCGCCT |  |  |
| *speM* (for.) | AGCACCTTCCTCTTTCTCGCCT | 600 | 1 |
| *speM* (rev.) | CAAAGTGACTTACTTTACTCATATCAATCG |  |  |
| **DNAse1:** |  |  |  |
| *spd1* (for.) | CCCTTCAGGATTGCTGTCAT | 400 | 2 |
| *spd1* (rev.) | ACTGTTGACGCAGCTAGGG |  |  |
| **Streptodornase** |  |  |  |
| *sdn* (for.) | ACCCCATCGGAAGATAAAGC | 489 | 3 |
| *sdn* (rev.) | AACGTTCAACAGGCGCTTAC |  |  |
| **Streptolysin S** |  |  |  |
| *sagA* (for.) | TACTTCAAATATTTTAGCTACT | 487 | 4 |
| *sagA* (rev.) | GATGATACCCCGATAAGGATAA |  |  |

**Reference:**

1. Rato MG, Nerlich A, Bergmann R, Bexiga R, Nunes SF, Vilela CL, Santos-Sanches I, Chhatwal GS. Virulence gene pool detected in bovine group *C Streptococcus dysgalactiae* subsp. *dysgalactiae* using a group A Streptococcus pyogenes virulence microarray. Journal of Clinical Microbiology. 2011; 49: 2470-2479.
2. Green NM, Beres SB, Graviss EA, Allison JE, McGeer AJ, Vuopio-Varkila J, LeFebvre RB, Musser JM. Genetic diversity among type emm28 group A Streptococcus strains causing invasive infections and pharyngitis. Journal of Clinical Microbiology. 2005; 43: 4083–4091.
3. Matsumoto M, Hoe NP, Liu M, Beres SB, Sylva GL, Brandt CM, Haase G, Musser JM. Intrahost sequence variation in the streptococcal inhibitor of complement gene in patients with human pharyngitis. The Journal of Infectious Diseases. 2003; 187: 604–612.
4. Abdelsalam M, Chen SC, Yoshida T. Dissemination of streptococcal pyrogenic exotoxin G (spegg) with an IS-like element in fish isolates of *Streptococcus dysgalactiae*. FEMS Microbiology Letters. 2010; 309: 105–113.
