# Supplementary material for: Streptococcus dysgalactiae subsp. dysgalactiae isolated from milk of the bovine udder as emerging pathogens: In vitro and in vivo infection of human cells and zebrafish as biological models
Source: Microbiologyopen. 2018 Mar 25;8(1):e00623. doi: 10.1002/mbo3.623 (PMC6341033; doi:10.1002/mbo3.623)
Supplement: Supplementary file 2 [file MBO3-8-e00623-s002.pdf]

```
                                      20                                  40                                  60
                                       |                                   |                                   |
VSD21+fbpA   ACAGATAAAT  TAAAACGGTT  CCGTGAGTTT  TTTGCTAGAC  CTACTCAAGC  AAGTTTAACC  60
 VSD5+fbpA   ACAGATAAAT  TAAAACGGTT  CCGTGAGTTT  TTTGCTAGAC  CTACTCAAGC  AAGTTTAACC  60
 VSD9+fbpA   ACAGATAAAT  TAAAACGGTT  CCGTGAGTTT  TTTGCTAGAC  CTACTCAAGC  AAGTTTAACC  60
VSD23+fbpA   ACAGATAAAT  TAAAACGGTT  CCGTGAGTTT  TTTGCTAGAC  CTACTCAAGC  AAGTTTAACC  60
VSD24+fbpA   ACAGATAAAT  TAAAACGGTT  CCGTGAGTTT  TTTGCTAGAC  CTACTCAAGC  AAGTTTAACC  60
VSD13+fbpA   ACAGATAAAT  TAAAACGGTT  CCGTGAGTTT  TTTGCTAGAC  CTACTCAAGC  AAGTTTAACC  60
COI289+fbpA  ACAGACAAAT  TAAAACGGTT  CCGTGAGTTT  TTTGCTAGAC  CTACTCAAGC  AAGTTTAACC  60
SDSE GGS124  ACAGACAAAT  TAAAACGGTT  CCGTGAGTTT  TTTGCTAGAC  CTACTCAAGC  AAGTTTAACC  60
HSM53+fbpA   ACAGACAAAT  TAAAACGGTT  CCGTGAGTTT  TTTGCTAGAC  CTACTCAAGC  AAGTTTAACC  60
 Consensus   ACAGATAAAT  TAAAACGGTT  CCGTGAGTTT  TTTGCTAGAC  CTACTCAAGC  AAGTTTAACC
   2,0bits
Sequence logo
   0,0bits
                                      80                                 100                                 120
                                       |                                   |                                   |
VSD21+fbpA   AATACCTCTT  TTGCACCAGT  TCTTTTCTCA  GAGAGCCAAG  CAACGTTTGA  GACTTTATCA  120
 VSD5+fbpA   AATACCTCTT  TTGCACCAGT  TCTTTTCTCA  GAGAGCCAAG  CAACGTTTGA  GACTTTATCA  120
 VSD9+fbpA   AATACCTCTT  TTGCACCAGT  TCTTTTCTCA  GAGAGCCAAG  CAACGTTTGA  GACTTTATCA  120
VSD23+fbpA   AATACCTCTT  TTGCACCAGT  TCTTTTCTCA  GAGAGCCAAG  CAACGTTTGA  GACTTTATCA  120
VSD24+fbpA   AATACCTCTT  TTGCACCAGT  TCTTTTCTCA  GAGAGCCAAG  CAACGTTTGA  GACTTTATCA  120
VSD13+fbpA   AATACCTCTT  TTGCACCAGT  TCTTTTCTCA  GAGAGCCAAG  CAACGTTTGA  GACTTTATCA  120
COI289+fbpA  AATACCTCTT  TTGCACCAGT  TCTTTTCTCA  GAGAGCCAAG  TGACGTTTGA  GACTTTATCT  120
SDSE GGS124  AATACCTCTT  TTGCACCAGT  TCTTTTCTCA  GAGAGCCAAG  TGACGTTTGA  GACTTTATCT  120
HSM53+fbpA   AATACCTCTT  TTGCACCAGT  TCTTTTCTCA  GAGAGTCAAG  TGACGTTTGA  GACTTTATCT  120
 Consensus   AATACCTCTT  TTGCACCAGT  TCTTTTCTCA  GAGAGCCAAG  CAACGTTTGA  GACTTTATCA
   2,0bits
Sequence logo
   0,0bits
                                     140                                 160                                 180
                                       |                                   |                                   |
VSD21+fbpA   GAGATGCTTG  ATCACTTTTA  TCAAGATAAA  GCTGAGCGTG  ACCGTATCAA  TCAACAAGCG  180
 VSD5+fbpA   GAGATGCTTG  ATCACTTTTA  TCAAGATAAA  GCTGAGCGTG  ACCGTATCAA  TCAACAAGCG  180
 VSD9+fbpA   GAGATGCTTG  ATCACTTTTA  TCAAGATAAA  GCTGAGCGTG  ACCGTATCAA  TCAACAAGCG  180
VSD23+fbpA   GAGATGCTTG  ATCACTTTTA  TCAAGATAAA  GCTGAGCGTG  ACCGTATCAA  TCAACAAGCG  180
VSD24+fbpA   GAGATGCTTG  ATCACTTTTA  TCAAGATAAA  GCTGAGCGTG  ACCGTATCAA  TCAGCAAGCA  180
VSD13+fbpA   GAGATGCTTG  ATCACTTTTA  TCAAGATAAA  GCTGAGCGTG  ACCGTATCAA  TCAGCAAGCG  180
COI289+fbpA  GAGATGCTTG  ATCACTTTTA  TCAAGATAAG  GCTGAGCGTG  ACCGTATCAA  TCAGCAAGCG  180
SDSE GGS124  GAGATGCTTG  ATCACTTTTA  TCAAGATAAG  GCTGAGCGTG  ACCGTATCAA  TCAGCAAGCG  180
HSM53+fbpA   GAGATGCTTG  ATCACTTTTA  TCAAGATAAG  GCTGAGCGTG  ACCGTATCAA  TCAGCAAGCG  180
 Consensus   GAGATGCTTG  ATCACTTTTA  TCAAGATAAA  GCTGAGCGTG  ACCGTATCAA  TCAGCAAGCG
   2,0bits
Sequence logo
   0,0bits
                                     200                                 220                                 240
                                       |                                   |                                   |
VSD21+fbpA   AGTGACTTGA  TTCACCGTGT  ACAAAATGAA  CTAGAAAAAA  ATCGCAACAA  ACTAAGAAAA  240
 VSD5+fbpA   AGTGACTTGA  TTCACCGTGT  ACAAAATGAA  CTAGAAAAAA  ATCGCAACAA  ACTAAGAAAA  240
 VSD9+fbpA   AGTGACTTGA  TTCACCGTGT  ACAAAATGAA  CTAGAAAAAA  ATCGCAACAA  ACTAAGAAAA  240
VSD23+fbpA   AGTGACTTGA  TTCACCGTGT  ACAAAATGAA  CTAGAAAAAA  ATCGCAACAA  ACTAAGAAAA  240
VSD24+fbpA   AGTGACTTGA  TTCACCGTGT  ACAAAATGAA  CTAGAAAAAA  ATCGCAACAA  ACTAAGAAAA  240
VSD13+fbpA   AGTGACTTGA  TTCATCGTGT  ACAAAATGAA  CTAGAAAAAA  ATCGCAACAA  ACTAAGAAAA  240
COI289+fbpA  AGTGACTTGA  TTCACCGTGT  GCAAAATGAA  CTAGACAAAA  ATCGCAACAA  ACTAAGTAAA  240
SDSE GGS124  AGTGACTTGA  TTCACCGTGT  GCAAAATGAA  CTAGACAAAA  ATCGCAACAA  ACTAAGTAAA  240
HSM53+fbpA   AGTGACTTGA  TTCACCGTGT  GCAAAATGAA  CTAGACAAAA  ATCGCAACAA  ACTAAGTAAA  240
 Consensus   AGTGACTTGA  TTCACCGTGT  ACAAAATGAA  CTAGAAAAAA  ATCGCAACAA  ACTAAGAAAA
   2,0bits
Sequence logo
   0,0bits
                                     260                                 280                                 300
                                       |                                   |                                   |
VSD21+fbpA   CAAGAAGCTG  AACTTTTGGC  TACGGAAAAC  GCTGAGTTGT  TCCGCCAAAA  AGGTGAGCTG  300
 VSD5+fbpA   CAAGAAGCTG  AACTTTTGGC  TACGGAAAAC  GCTGAGTTGT  TCCGCCAAAA  AGGTGAGCTG  300
 VSD9+fbpA   CAAGAAGCTG  AACTTTTGGC  TACGGAAAAC  GCTGAGTTGT  TCCGCCAAAA  AGGTGAGCTG  300
VSD23+fbpA   CAAGAAGCTG  AACTTTTGGC  TACGGAAAAC  GCTGAGTTGT  TCCGCCAAAA  AGGTGAGCTG  300
VSD24+fbpA   CAAGAAGCTG  AACTTTTGGC  TACGGAAAAC  GCTGAGTTGT  TCCGCCAAAA  AGGTGAGCTG  300
VSD13+fbpA   CAAGAAGCTG  AACTTTTGGC  TACGGAAAAC  GCTGAGTTGT  TCCGCCAAAA  AGGTGAGCTG  300
COI289+fbpA  CAAGAAGCTG  AACTTTTGGC  TACGGAAAAC  GCTGAGTTGT  TCCGTCAAAA  AGGAGAGCTG  300
SDSE GGS124  CAAGAAGCTG  AACTTTTGGC  TACGGAAAAC  GCTGAGTTGT  TCCGTCAAAA  AGGAGAGCTG  300
HSM53+fbpA   CAAGAAGCTG  AACTTTTGGC  TACGGAAAAC  GCTGAGTTGT  TCCGTCAAAA  AGGAGAGCTG  300
 Consensus   CAAGAAGCTG  AACTTTTGGC  TACGGAAAAC  GCTGAGTTGT  TCCGCCAAAA  AGGTGAGCTG
   2,0bits
Sequence logo
   0,0bits
                                     320                                 340                                 360
                                       |                                   |                                   |
VSD21+fbpA   TTAACCACTT  ACCTCAGTAT  GGTTCCTAAT  AACCAGGACT  CCGTGGTGTT  AGACAATTAT  360
 VSD5+fbpA   TTAACCACTT  ACCTCAGTAT  GGTTCCTAAT  AACCAGGACT  CCGTGGTGTT  AGACAATTAT  360
 VSD9+fbpA   TTAACCACTT  ACCTCAGTAT  GGTTCCTAAT  AACCAGGACT  CCGTGGTGTT  AGACAATTAT  360
VSD23+fbpA   TTAACCACTT  ACCTCAGTAT  GGTTCCTAAT  AACCAGGACT  CCGTGGTGTT  AGACAATTAT  360
VSD24+fbpA   TTAACCACTT  ACCTCAGTAT  GGTTCCTAAT  AACCAGGACT  CCGTGGTGTT  AGACAATTAT  360
VSD13+fbpA   TTAACCACTT  ACCTCAGTAT  GGTTCCTAAT  AACCAGGACT  CCGTGGTGTT  AGACAATTAT  360
COI289+fbpA  TTAACCACTT  ACATGAGTAT  GGTTCCTAAT  AACCAAGACT  CCGTGGTATT  AGACAATTAT  360
SDSE GGS124  TTAACCACTT  ACATGAGTAT  GGTTCCTAAT  AACCAAGACT  CCGTGGTATT  AGACAATTAT  360
HSM53+fbpA   TTAACCACTT  ACATGAGTAT  GGTTCCTAAT  AACCAAGACT  CCGTGGTATT  AGACAATTAT  360
 Consensus   TTAACCACTT  ACCTCAGTAT  GGTTCCTAAT  AACCAGGACT  CCGTGGTGTT  AGACAATTAT
   2,0bits
Sequence logo
   0,0bits
```

```
                                         380                                    400                                    420
VSD21+fbpA   TACACAGGCG  AAAAGATTGA  GATTGCCTTA  GACAAGGCAC  TAACACCCAA  TCAAAATGCT  420
VSD5+fbpA    TACACAGGCG  AAAAGATTGA  GATTGCCTTA  GACAAGGCAC  TAACACCCAA  TCAAAATGCT  420
VSD9+fbpA    TACACAGGCG  AAAAGATTGA  GATTGCCTTA  GACAAGGCAC  TAACACCCAA  TCAAAATGCT  420
VSD23+fbpA   TACACAGGCG  AAAAGATTGA  GATTGCCTTA  GACAAGGCAC  TAACACCCAA  TCAAAATGCT  420
VSD24+fbpA   TACACAGGCG  AAAAGATTGA  GATTGCCTTA  GACAAGGCAC  TAACACCCAA  TCAAAATGCT  420
VSD13+fbpA   TACACAGGCG  AAAAGATTGA  GATTGCCTTA  GACAAGGCAC  TAACACCCAA  TCAAAATGCT  420
COI289+fbpA  TACACGGGCG  AAAAGCTTGA  GATTGCCCTA  GACAAGGCAC  TAACACCTAA  TCAAAATGCT  420
SDSE GGS124  TACACGGGCG  AAAAGCTTGA  GATTGCCCTA  GACAAGGCAC  TAACACCTAA  TCAAAATGCT  420
HSM53+fbpA   TACACGGGCG  AAAAGCTTGA  AATTGCCCTA  GACAAGGCAC  TAACGCCTAA  TCAAAATGCT  420
Consensus    TACACAGGCG  AAAAGATTGA  GATTGCCTTA  GACAAGGCAC  TAACACCCAA  TCAAAATGCT
Sequence logo (2,0bits / 0,0bits)

                                         440                                    460                                    480
VSD21+fbpA   CAACGTTATT  TTAAAAAATA  CCAAAAGCTA  AAAGAAGCTG  TCAAACACTT  ATCTGGATTG  480
VSD5+fbpA    CAACGTTATT  TTAAAAAATA  CCAAAAGCTA  AAAGAAGCTG  TCAAACACTT  ATCTGGATTG  480
VSD9+fbpA    CAACGTTATT  TTAAAAAATA  CCAAAAGCTA  AAAGAAGCTG  TCAAACACTT  ATCTGGATTG  480
VSD23+fbpA   CAACGTTATT  TTAAAAAATA  CCAAAAGCTA  AAAGAAGCTG  TCAAACACTT  ATCTGGATTG  480
VSD24+fbpA   CAACGTTATT  TTAAAAAATA  CCAAAAGCTA  AAAGAAGCTG  TCAAACACTT  ATCTGGATTG  480
VSD13+fbpA   CAACGTTATT  TTAAAAAATA  CCAAAAGCTA  AAAGAAGCTG  TCAAACACTT  ATCTGGATTG  480
COI289+fbpA  CAACGTTATT  TTAAAAAATA  CCAAAAGCTA  AAAGAAGCTG  TCAAACACTT  ATCTGGATTG  480
SDSE GGS124  CAACGTTATT  TTAAAAAATA  CCAAAAGCTA  AAAGAAGCTG  TCAAACACTT  ATCTGGATTG  480
HSM53+fbpA   CAACGTTATT  TTAAAAAATA  CCAAAAGCTA  AAAGAAGCTG  TCAAACACTT  ATCTGGATTG  480
Consensus    CAACGTTATT  TTAAAAAATA  CCAAAAGCTA  AAAGAAGCTG  TCAAACACTT  ATCTGGATTG
Sequence logo (2,0bits / 0,0bits)

                                         500                                    520                                    540
VSD21+fbpA   ATTACCGATA  CCAAGCAAAG  TATTGCGTAT  TTTGAGAGTG  TGGACTACAA  TTTGTCCCAA  540
VSD5+fbpA    ATTACCGATA  CCAAGCAAAG  TATTGCGTAT  TTTGAGAGTG  TGGACTACAA  TTTGTCCCAA  540
VSD9+fbpA    ATTACCGATA  CCAAGCAAAG  TATTGCGTAT  TTTGAGAGTG  TGGACTACAA  TTTGTCCCAA  540
VSD23+fbpA   ATTACCGATA  CCAAGCAAAG  TATTGCGTAT  TTTGAGAGTG  TGGACTACAA  TTTGTCCCAA  540
VSD24+fbpA   ATTACCGATA  CCAAGCAAAG  TATTGCGTAT  TTTGAGAGTG  TGGACTACAA  TTTGTCCCAA  540
VSD13+fbpA   ATTACCGATA  CCAAGCAAAG  TATTGCGTAT  TTTGAGAGTG  TGGACTACAA  TTTGTCCCAA  540
COI289+fbpA  ATTGCCGATA  CCAAGCAAAG  TATTGCTTAC  TTTGAGAGCG  TGGACTACAA  TTTATCCCAA  540
SDSE GGS124  ATTGCCGATA  CCAAGCAAAG  TATTGCTTAC  TTTGAGAGCG  TGGACTACAA  TTTATCCCAA  540
HSM53+fbpA   ATTGCCGATA  CCAAGCAAAG  TATTGCTTAC  TTTGAGAGCG  TGGACTACAA  TTTATCCCAA  540
Consensus    ATTACCGATA  CCAAGCAAAG  TATTGCGTAT  TTTGAGAGTG  TGGACTACAA  TTTGTCCCAA
Sequence logo (2,0bits / 0,0bits)

                                         560                                    580                                    600
VSD21+fbpA   GTAAGTATCG  AAGATATTGA  AGATATTCGG  GAAGAGTTAT  ATCAAGCTGG  CTTTTTGAAA  600
VSD5+fbpA    GTAAGTATCG  AAGATATTGA  AGATATTCGG  GAAGAGTTAT  ATCAAGCTGG  CTTTTTGAAA  600
VSD9+fbpA    GTAAGTATCG  AAGATATTGA  AGATATTCGG  GAAGAGTTAT  ATCAAGCTGG  CTTTTTGAAA  600
VSD23+fbpA   GTAAGTATCG  AAGATATTGA  AGATATTCGG  GAAGAGTTAT  ATCAAGCTGG  CTTTTTGAAA  600
VSD24+fbpA   GTAAGTATCG  AAGATATTGA  AGATATTCGG  GAAGAGTTAT  ATCAAGCTGG  CTTTTTGAAA  600
VSD13+fbpA   GTAAGTATCG  AAGATATTGA  AGATATTCGG  GAAGAGTTAT  ATCAAGCTGG  CTTTTTGAAA  600
COI289+fbpA  GCAAGTATCG  AAGATATTGA  AGATATTCGG  GAAGAGTTAT  ATCAGGCTGG  CTTTTTGAAG  600
SDSE GGS124  GCAAGTATCG  AAGATATTGA  AGATATTCGG  GAAGAGTTAT  ATCAGGCTGG  CTTTTTGAAG  600
HSM53+fbpA   GCAAGTATCG  AAGATATTGA  AGATATTCGG  GAAGAGTTAT  ATCAGGCTGG  CTTTTTGAAG  600
Consensus    GTAAGTATCG  AAGATATTGA  AGATATTCGG  GAAGAGTTAT  ATCAAGCTGG  CTTTTTGAAA
Sequence logo (2,0bits / 0,0bits)

                                         620
VSD21+fbpA   AGTCGCCACC  GTGATAAACG  620
VSD5+fbpA    AGTCGCCACC  GTGATAAACG  620
VSD9+fbpA    AGTCGCCACC  GTGATAAACG  620
VSD23+fbpA   AGTCGCCACC  GTGATAAACG  620
VSD24+fbpA   AGTCGCCACC  GTGATAAACG  620
VSD13+fbpA   AGTCGCCACC  GTGATAAACG  620
COI289+fbpA  AGTCGCCACC  GTGATAAACG  620
SDSE GGS124  AGTCGCCACC  GTGATAAACG  620
HSM53+fbpA   AGTCGCCACC  GTGATAAACG  620
Consensus    AGTCGCCACC  GTGATAAACG
Sequence logo (2,0bits / 0,0bits)
```
